# Mining traits for the enrichment and isolation of not-yet-cultured populations

**DOI:** 10.1186/s40168-019-0708-4

**Published:** 2019-06-25

**Authors:** An-Ni Zhang, Yanping Mao, Yubo Wang, Tong Zhang

**Affiliations:** 1 Environmental Biotechnology Laboratory, The University of Hong Kong, Hong Kong, China; 2 College of Chemistry and Environmental Engineering, Shenzhen University, Shenzhen, China

**Keywords:** Enrichment and isolation, Bioinformatic pipeline, Pan-genome analysis, Metabolisms, Not-yet-cultured bacteria

## Abstract

**Background:**

The lack of pure cultures limits our understanding into 99% of bacteria. Proper interpretation of the genetic and the transcriptional datasets can reveal clues for the enrichment and even isolation of the not-yet-cultured populations. Unraveling such information requires a proper mining method.

**Results:**

Here, we present a method to infer the hidden traits for the enrichment of not-yet-cultured populations. We demonstrate this method using *Candidatus* Accumulibacter. Our method constructs a whole picture of the carbon, electron, and energy flows in the not-yet-cultured populations from the genomic datasets. Then, it decodes the coordination across three flows from the transcriptional datasets. Based on it, our method diagnoses the status of the not-yet-cultured populations and provides strategy to optimize the enrichment systems.

**Conclusion:**

Our method could shed light to the exploration into the bacterial dark matter in the environments.

**Electronic supplementary material:**

The online version of this article (10.1186/s40168-019-0708-4) contains supplementary material, which is available to authorized users.

## Background

Genomes of a phylogenetic lineage hold the information of the function potentials and ecological adaptations, which can provide hints for its enrichment and isolation [1, 2]. Unraveling such information requires a proper tool. For genetic analysis, pan-genome is wildly used to characterize the key features of a population [3, 4]. However, pan-genome analysis is originally designed for complete genomes [5], while most genomes of the not-yet-cultured populations are incomplete. To apply pan-genome to these non-yet-cultured populations, we need a novel approach for the incomplete genomes.

Genomic information alone may not be sufficient to provide such traits. Still, many important populations are not-yet-cultured even with available genomes and the knowledge of their optimal niches [6]. Taking *Candidatus* Accumulibacter (Accumulibacter) as an example, as the primary functional population in the enhanced biological phosphorus removal (EBPR), 13 Accumulibacter genomes have been retrieved and analyzed [7–12]. Besides, its optimal enrichment conditions have been well studied for more than two decades [13–15]. However, the lack of pure cultures is still limiting our understanding. One critical problem is that different lineages of Accumulibacter display diverse niche adaptations of, i.e., salinity, carbon sources, and electron acceptors [11, 16–18]. Thus, even though we can use the same optimal conditions from previous studies to enrich the Accumulibacter, it may not be favorable for the specific strains inside.

Combining the genetic information with the transcriptional analysis can provide the clues to enrich the strains in our systems. The transcriptional analysis has been conducted on Accumulibacter [19–21], but mainly focusing on the separate pathways of carbon (C), nitrogen (N), phosphorus (P), and sulfur (S). Instead, if we look at the whole picture of the coordination among pathways in response to the environmental conditions [22], we could differentiate the optimal and suboptimal status of a population. This highlights the need of a tool to mine the whole picture from the transcriptional patterns and link it to the environmental conditions.

In this study, we present a method to decode the hidden traits from genetic and transcriptional datasets, for guiding the enrichment of not-yet-cultured bacterial populations. We use Accumulibacter as a demonstration for both anaerobic (AN) and aerobic (AE) organotrophs and chemoheterotrophs. Its biochemical complexity and significance to environmental engineering make it an example of both interest and importance.

## Materials and methods

### Genomic analysis

Pan-genome analysis designed in this study classifies the key features of a phylogenetic lineage using the complete/incomplete genomes. The pan-genome is defined as a whole set of non-orthologous genes in all available genomes (total number is *N*) of a phylogenetic lineage [23]. All non-orthologous genes are subdivided into core-, dispensable-, and strain-specific genomes based on the frequency of their occurrence in *N* genomes. Previously, core-genome is defined as genes shared by all genomes [4, 5]. However, because of the incomplete (draft) genomes, such a strict definition will result in the low coverage of core-genome. Thus, we propose an approach for pan-genome subdivision by evaluating the false-negative (FN) and false-positive (FP) rates. Core-genome is defined as genes shared by at least *n* genomes (*n* ≤ *N*) when all FN and FP rates are less than 1% (Additional file 1: Supplementary information and Table S1). Based on this cutoff *n*, the pan-genome is subdivided into core-, dispensable-, and strain-specific genomes as the collection of common genes shared by at least *n* genomes, accessory genes shared by a subset (2 to *n*-1 genomes), and unique genes of one genome, respectively. The coverage (100%—FN) and the accuracy (100%—FP) of core-, dispensable-, and strain-specific genomes are maintained as 99%.

The representativeness of the Accumulibacter genomes is illustrated by gene occurrence distribution and pan-genome sampling curves. The occurrence frequency of a gene is calculated by counting its orthologous genes in all *N* genomes (Additional file 1: Table S2) [5, 24]. This occurrence frequency is used to subdivide the pan-genome and to generate the pan-genome sampling curves. The size of core-, strain-specific, and pan-genomes is predicted by fitting exponential decaying functions, with each addition of a new genome [23, 25].

### A new metabolism framework: construct a whole picture

First of all, the metabolic pathways of pan-genome are annotated. We define the pan-pathway as all the non-redundant functions encoded by the pan-genome. The pan-genome represents all genotypes (sequences) meanwhile the pan-pathway represents all phenotypes (functions) of a population. The Accumulibacter pan-genome is annotated by KEGG (21st November 2016) [26] and eggNOG 4.5 databases [27, 28] to construct the Accumulibacter pan-pathway. The pan-pathway is also subdivided into core-, dispensable-, and strain-specific pathways as the collections of common functions shared by at least *n* genomes, accessory functions shared by a subset (2 to *n*-1 genomes), and unique functions of one genome, respectively. The split pathways are summarized into functional modules, such as glycolysis and tricarboxylic acid cycle (TCA cycle).

We construct a novel metabolism framework to assess the main role of a module in the carbon, electron, and energy flows. Here, the carbon flow refers to the fundamental organic and inorganic carbon metabolism, the electron flow refers to the redox reactions between electron donors and electron acceptors, and the energy flow refers to the generation and consumption ATP. It assigns the main role to a module by evaluating its contribution to the carbon, electron, and energy flows as sources or consumers (Table 1). Then, it distinguishes the primary sources and consumers from the secondary sources and consumers. For example, it first assigns the main role of glycolysis as carbon-providing and electron-providing. Then, it distinguishes that the glycolysis is a primary electron-providing module for Accumulibacter in AN phase (Table 2).

**Table 1:**
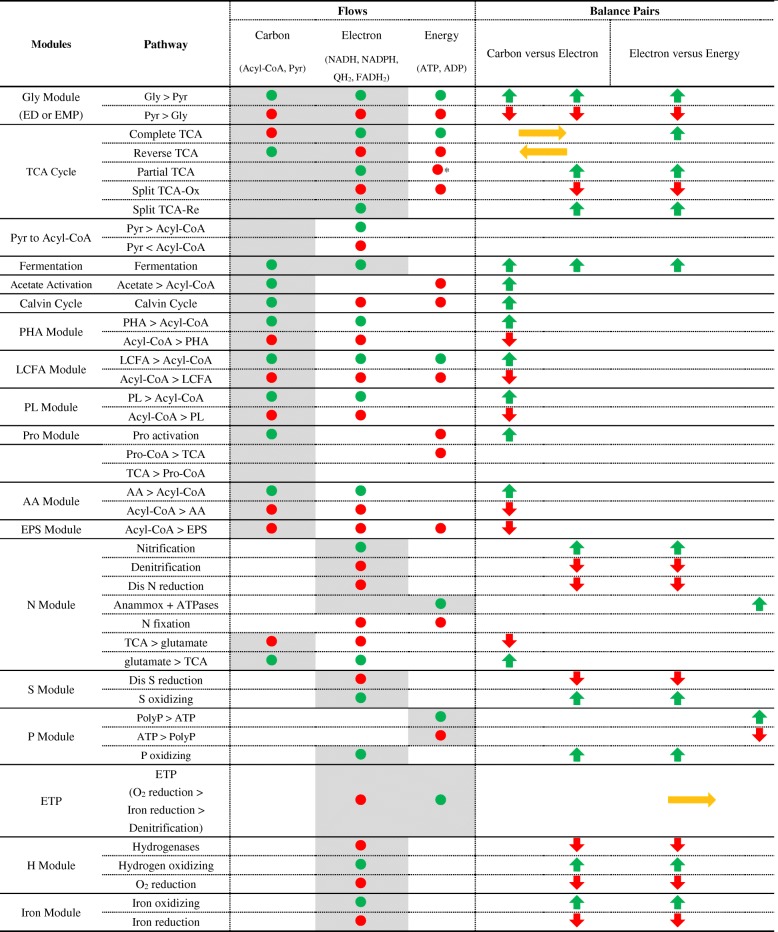
The main roles (shades) and contribution (nodes) of functional modules to carbon, electron, and energy flows

Functional modules could be employed by their main flows to adapt to the unbalanced status of two balance pair (carbon flow versus electron flow and electron flow versus energy flow). The color and size of the nodes and arrows represented the amount and direction (green, providing; red, consuming; yellow, interconverting) of the contribution to each flow

*Carbon flow from partial TCA cycle to Pro module

**Table 2:**
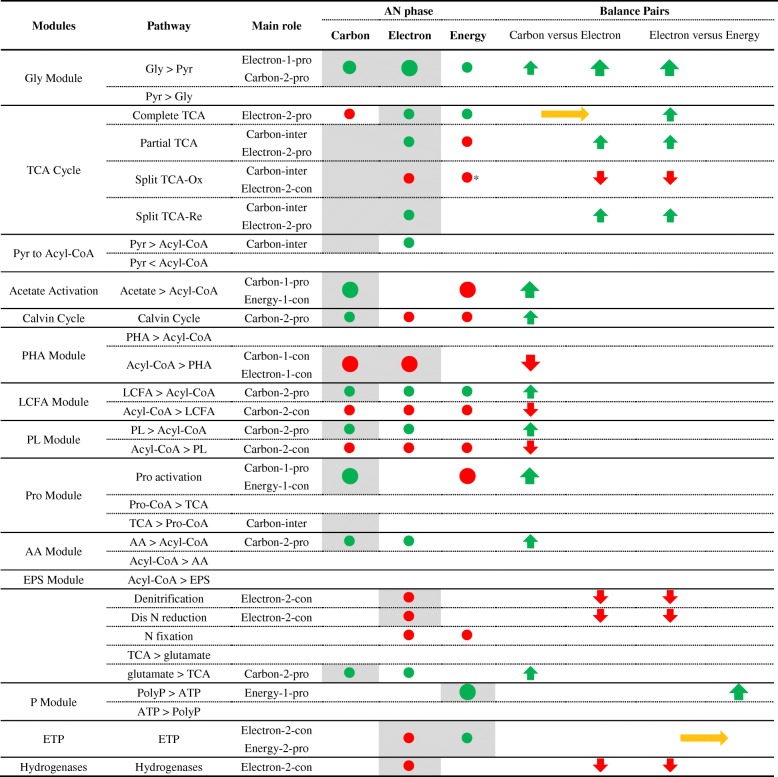
The main roles (shades) and contribution (nodes) of Accumulibacter pan-pathway to carbon, electron, and energy flows in AN phase

Functional modules could be employed by their main flows to adapt to the unbalanced status of two balance pair (carbon flow versus electron flow and electron flow versus energy flow). The color and size of the nodes and arrows represented the amount and direction (green, providing; red, consuming; yellow, interconverting) of the contribution to each flow. *1-pro* primary providing, *2-pro* secondary providing, *1-con* primary consuming, *2-con* secondary consuming, *inter* interconverting

*Carbon flow from partial TCA cycle to Pro module

### A new transcriptomic analysis: diagnose the status

The balance among the primary carbon, electron, and energy sources is the key feature to differentiate the optimal and suboptimal conditions. These three sources are linked by the electron flow as two balance pair (carbon flow versus electron flow and electron flow versus energy flow). The carbon flow is motivated by redox reactions from primary sources to primary consumers. The electron flow is driven by the cellular redox and the electron transport phosphorylation (ETP) to recycle electron carriers [29], balance redox condition, and convert energy. Within the energy flow, the energy-providing and energy-consuming rates [30] are well balanced [31] for the equilibrium of ATP/ADP. Overall, the primary electron sources are competed by the primary carbon sources, primary energy consumers, and redox balance.

The varying status of two balance pair (carbon flow versus electron flow and electron flow versus energy flow) represents the status of a population. Each balanced pair has three types (i.e., primary carbon source is excessive, balanced, or insufficient than primary electron source), which results in totally nine scenarios to represent all the status. We use transcriptional data to evaluate these two balance pairs, to diagnose the status, and to optimize the enrichment conditions. When the two balance pairs are balanced (the optimal status), the transcriptional pattern mainly involves the fundamental pathways (primary sources and consumers) for the most effective growth. However, the disruption of any balance pair may result in the coordination of secondary sources and consumers to help balance the pair for a more effective growth.

### Pipeline demonstration

We summarize the above methods into a bioinformatic pipeline called Pan-genome and Pan-pathway Pipeline (PAPP) (Additional file 1: Supplementary information and Figure S1). PAPP is demonstrated by 13 Accumulibacter genomes (Additional file 1: Table S2) and two available metatranscriptomic datasets of EBPR studies (IMG/M-3300002341-3300002346, NCBI-SRP038016). It constructs the Accumulibacter pan-genome and pan-pathway. It transforms the metatranscriptomic datasets to cellular relative transcriptional activity (CRPKM) (Additional file 2: Table S3). Then, it visualizes the Accumulibacter pan-pathway and the transcriptional dynamics by using Cytoscape 3.3.0 [32]. Based on the transcriptional patterns, it diagnoses the status of Accumulibacter in two enrichment conditions. The PAPP pipeline is available on https://github.com/caozhichongchong/PAPP.

## Results and discussion

### Accumulibacter pan-genome: complete representativeness

**Fig. 1:**
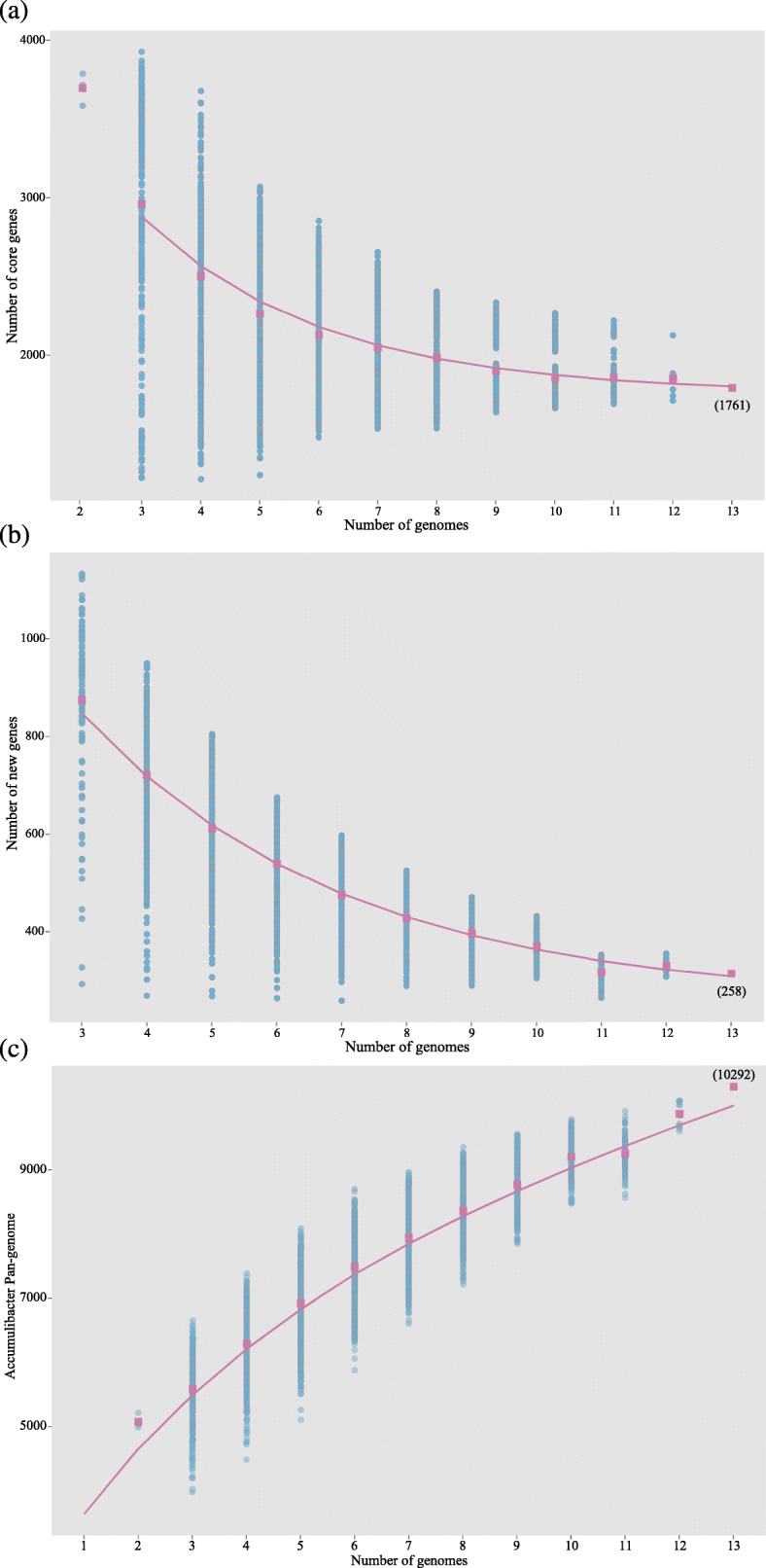
Reconstructed sampling curves of 13 Accumulibacter genomes by the exhausted subsampling method. The number of genes was calculated as a function of adding an *n*^th^ genome into the (*n*-1) genomes. The total number (≤ TN) of effective permutation for each *n* was represented by the number of circles, which were obtained by different genome combinations. **a** Core-genome sampling curve. The average number of core genes at each *n* number was plotted as squares, and the continuous curve represented the least-squares fit of the function $\mathrm{Fc}=\mathrm{Kc}\ \exp \left[-\frac{n}{\uptau \mathrm{c}}\right]+\Omega$. The best hit vector for Kc, τc, and Ω was 3000, 3.04, and 1761 with correlation *r*^2^ 0.98. **b** Strain-specific genome sampling curve. The average number of strain-specific genes at each *n* number was plotted as squares, and the continuous curve represented the least-squares fit of the function $\mathrm{Fs}=\mathrm{Ks}\ \exp \left[-\frac{n}{\uptau \mathrm{s}}\right]+\mathrm{tg}\left(\uptheta \right)$. The best hit vector for Ks, τs, and tg(θ) was 1234, 4.05, and 258 with correlation *r*^2^ 1.00. **c** Pan-genome sampling curve. The average number of all genes (size of pan-genome) at each *n* number was plotted as squares, and the continuous curve represented the least-squares fit of the function $P(n)=D+\mathrm{tg}\left(\uptheta \right)\left[n-1\right]+\mathrm{Ksexp}\left[-\frac{2}{\uptau \mathrm{s}}\right]\frac{1-\exp \left[-\frac{n-1}{\uptau \mathrm{s}}\right]}{1-\exp \left[-\frac{1}{\uptau \mathrm{s}}\right]}$. With the best hit vector 1234, 4.05, and 258 for Ks, τs, and tg(θ) of strain-specific genome fitting and *D* as 3634; the correlation *r*^2^ of pan-genome fitting is 1.00

The Accumulibacter genomes can completely represent the core-genome of Accumulibacter. The cutoff of Accumulibacter core-genome is 9 of 13 (Additional file 1: Tables S1 and S4). By the occurrence frequency of all genes (Additional file 1: Figure. S2), Accumulibacter pan-genome is subdivided into 21% core genes, 40% dispensable genes, and 39% strain-specific genes. The composition of pan-genome in this study shifted to the core-genome side compared to a previous study [33], which uses a strict criterion for orthologs. The size of core-genome drops and gradually reaches the plateau of 1761 (Ω) genes (Fig. 1a), and it would not decrease even with new genomes in the future. However, new genomes will continuously provide new strain-specific genes (Fig. 1b), supplementing 258 genes (tg(θ)) per genome (2.5% of Accumulibacter pan-genome). It indicates that Accumulibacter pan-genome would keep increasing with new genomes, although not significantly. This also suggested that even with a larger genome collection in the future, it would be impossible to cover all the diversity within the Accumulibacter population.

### Accumulibacter pan-pathway: the whole picture of its functions

**Fig. 2:**
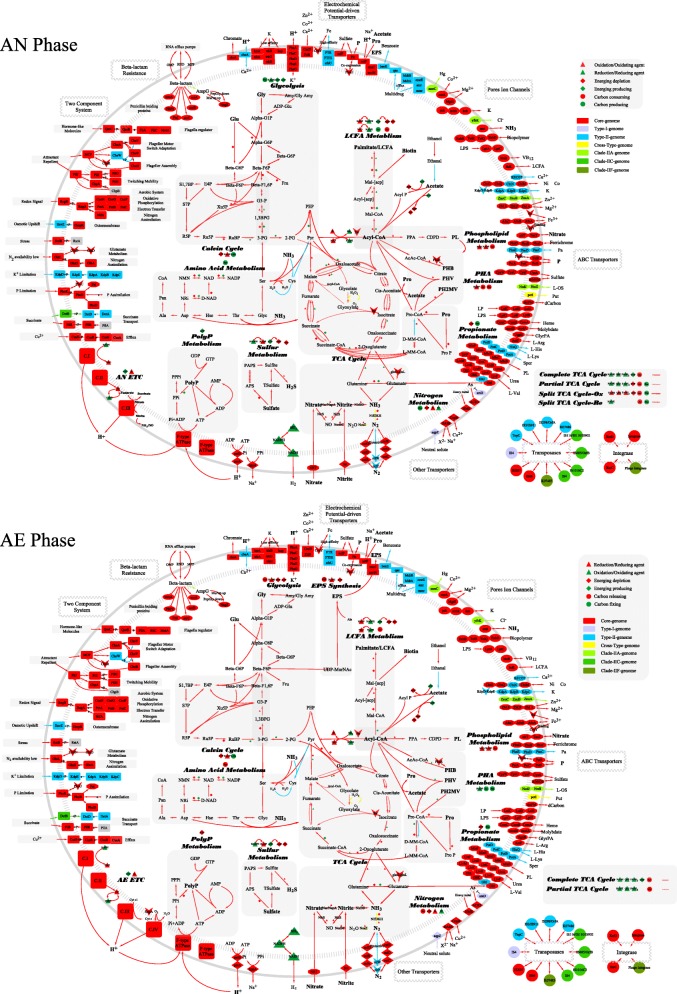
PAO pan-genome metabolic and non-metabolic model of core genes, dispensable genes, and unique genes. The core- (red), type I dispensable- (purple), Type II dispensable- (blue), cross-type dispensable- (yellow), and strain-specific (others) pathways were specifically highlighted by different colors. The main metabolic modules in Accumulibacter had different patterns and directions during AN and AE phases in the EBPR process. The carbon, electron, and energy flow of Accumulibacter pan-pathway were specifically demonstrated and discussed in two phases. The providers/consumers and the carrier form of each flow were distinguished by shapes and colors. The abbreviations were listed in the list of abbreviations and Additional file 1: Supplementary information

**Fig. 3:**
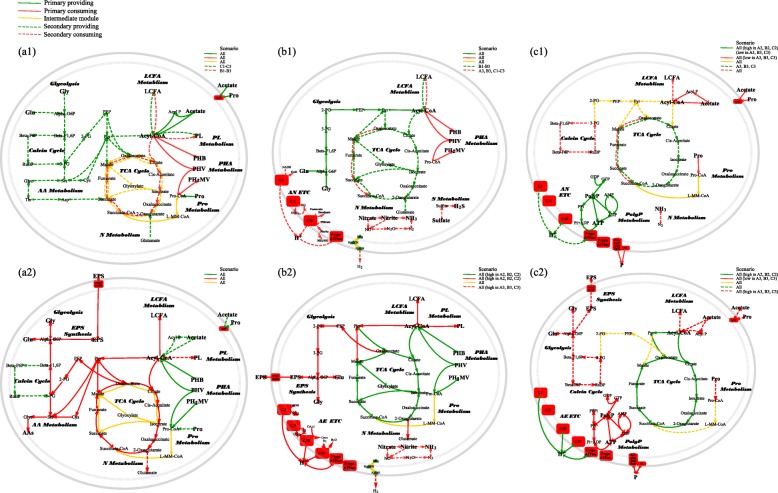
Simplified Accumulibacter pan-pathway separated into carbon, electron, and energy flows of EBPR anaerobic (AN) and aerobic (AE) phases. The variations of behavior represented by each scenario were specifically labeled for each flow, referring to the expression and activity (high and low) of related modules. **a1** Carbon flow in AN phase. **a2** Carbon flow in AE phase. **b1** Electron flow in AN phase. **b2** Electron flow in AE phase. **c1** Energy flow in AN phase. **c2** Energy flow in AE phase. The abbreviations of modules and chemical components are listed in the list of abbreviations and Additional file 1: Supplementary information

All lineages of Accumulibacter have one consistent core-pathway that covers all key functions involved in EBPR. The Accumulibacter pan-pathway contains 1676 functions, including 78% core functions, 18% dispensable functions, and 4% strain-specific functions. We present a whole picture of Accumulibacter pan-pathway (Fig. 2) in both AN and AE phases. The main role of each module including the three modes of TCA cycle [34] is assessed (Tables 2 and 3; Additional file 3: Table S5 and Additional file 4: Table S6) based on the metabolisms [7] and kinetic analysis [33]. We find out that all the primary pathways in carbon, electron, and energy flows (solid edges in Fig. 3) are completely accomplished by one consistent core-pathway (red edges in Fig. 2). It indicates that the Accumulibacter core-genome has a complete coverage for the EBPR functions. For Accumulibacter, the primary carbon, electron, and energy sources in AN phase are acetate, glycogen, and polyphosphorus (PolyP) respectively; and in AE phase are polyhydroxyalkanoates (PHA), PHA, and oxygen. Different sources in two phases result in different primary flows, and the continuous switching of two phases has an accumulative effect on the status of Accumulibacter. A brief summary of Accumulibacter pan-pathway is described below, with a complete description in Additional file 1: Supplementary information.

**Table 3:**
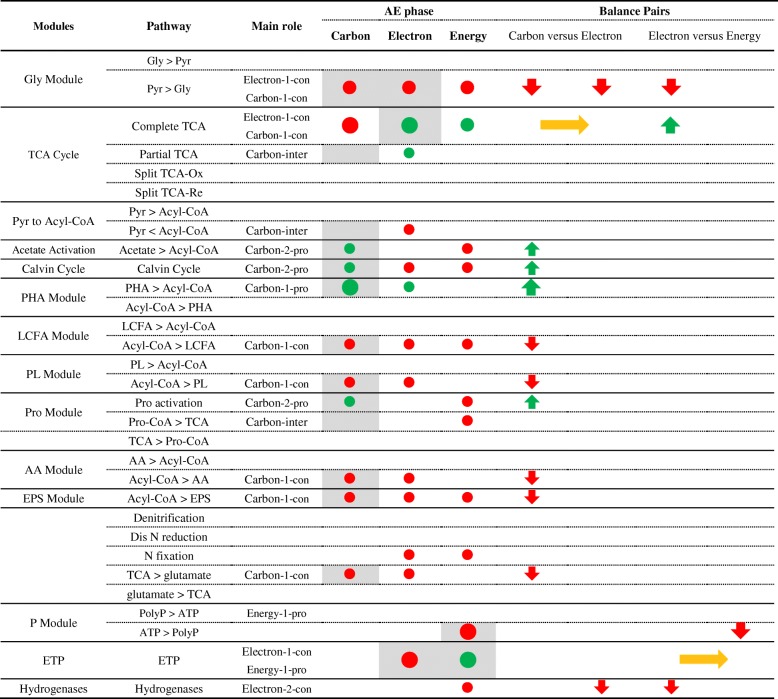
The main roles (shades) and contribution (nodes) of Accumulibacter pan-pathway to carbon, electron, and energy flows in AN phase

Functional modules could be employed by their main flows to adapt to the unbalanced status of two balance pairs (carbon flow versus electron flow and electron flow versus energy flow). The color and size of the nodes and arrows represented the amount and direction (green, providing; red, consuming; yellow, interconverting) of the contribution to each flow. *1-pro* primary providing, *2-pro* secondary providing, *1-con* primary consuming, *2-con* secondary consuming, *inter* interconverting

#### Carbon flow

Carbon flows from short-chain fatty acids (SCFAs) to PHA in AN phase. Accumulibacter can root and converge multiple carbon sources to acetyl-CoA (Acyl-CoA) as the hub of carbon flow for further allocation. Primarily, it uses SCFAs as the primary carbon source in AN phase, such as acetate or propionate (Pro), which is transported by *actP* and activated to Acyl-CoA and propionyl-CoA (Pro-CoA) (Fig. 3a1 and Table 2). It can also use the modules of glycolysis/gluconeogenesis (Gly), long-chain fatty acid (LCFA), amino acid (AA), nitrogen (glutamate), and Calvin cycle to provide secondary carbon sources [35]. Carbon is primarily consumed in by PHA module in AN phase, and them by secondary modules of complete TCA cycle, phospholipid (PL), and LCFA. In contrast to split and partial TCA cycles (transferring Acyl-CoA to PHA), complete TCA cycle supplements electrons at the cost of Acyl-CoA.

Carbon flows from PHA to complete TCA cycle in AE phase. Accumulibacter uses PHA as the main carbon source to feed the complete TCA cycle for electrons and energy in AE phase (Fig. 3a2 and Table 3). Accumulibacter employs partial TCA cycle to partition the carbon flow for glycogen generation by shunting the decarboxylation steps of complete TCA cycle [36]. Accumulibacter also invests carbon to LCFA, PL, AA, and exopolysaccharide (EPS) for cell synthesis.

#### Electron flow

Accumulibacter has flexible modules to maintain redox condition in AN phase. In AN phase, Gly module provides the primary electrons (electron donors) for Accumulibacter (Fig. 3b1). Moreover, complete, partial, and split TCA (reductive branch) cycles and LCFA modules also supplement electrons. The electrons are consumed to synthesize PHA [37, 38]. Like most assimilatory metabolisms, PHA synthesis requires NADPH, while the electrons available are mainly in other forms (NADH, fdH_2_, FADH_2_, and QH_2_). Thus, it is crucial for Accumulibacter to maintain the balance between the electron generation and transformation (transhydrogenases). To do that, Accumulibacter recruits the modules of TCA cycle, N modules (denitrification), ETP, and hydrogenases. ETP is proposed possible in AN phase with cytochrome *b/b6* oxidase [7], using nitrate, nitrite, and fumarate as terminal electron acceptors (TEAs) [39, 40]. Thus, when Accumulibacter has excessive electrons, these flexible modules could be activated to consume electrons at AN ending to maintain the recycle of electron carriers.

Accumulibacter uses partial TCA cycle to control the carbon, electron, and energy flows. In AE phase, electrons are released from Acyl-CoA (Fig. 3b2) through complete TCA cycle. Meanwhile, PHA and partial TCA cycle can release electrons. These electrons are mainly used by ETP for energy generation and by Gly module for glycogen production. Although partial TCA cycle has lower efficiency of electron generation compared to complete TCA cycle, partial TCA cycle is used to shunt the carbon flow to Gly. The flexibility between partial and complete TCA cycles to control the carbon, electron, and energy flows is a crucial ecological benefit endorsed by Accumulibacter.

#### Energy flow

The usage of PolyP as the energy source adapts Accumulibacter to the cycles of AN and AE environments. PolyP provides the energy for Accumulibacter to compete and store carbon sources in AN phase (Fig. 3c1). The energy is used to transport and activate acetate. In AE phase, the PolyP is recharged using the energy generated by ETP (Fig. 3c2).

### The status of Accumulibacter: one good example versus one bad example

**Fig 4.**
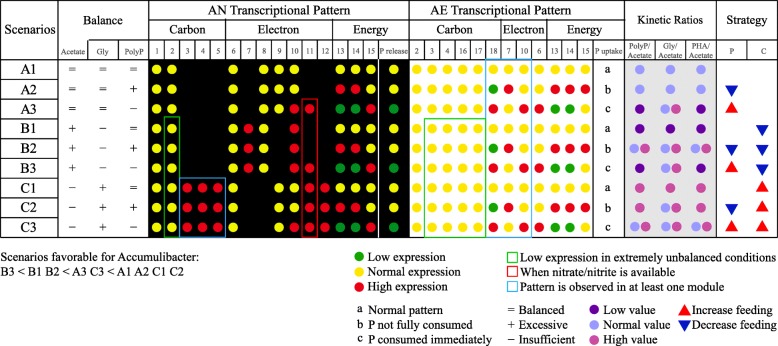
Nine scenarios of the behavior of Accumulibacter (metatranscriptomic pattern and chemical parameters) in response to all environmental conditions (balance of carbon, electron, and energy flows) with specific strategies proposed to balance the carbon and phosphorus feeding to optimize reactor operation. Number labels of modules: 1, acetate uptake; 2, PHA module; 3, LCFA module; 4, AA module; 5, glutamine/glutamate in *N* module; 6, Gly module; 7, complete TCA cycle; 8, reductive branch of split TCA cycle; 9, oxidative branch of split TCA cycle; 10, partial TCA cycle; 11, denitrification and dissimilatory nitrogen reduction in *N* module; 12, hydrogenases; 13, PolyP module; 14, phosphorus transporters (*pst* and *pit*); 15, ETP; 16, PL module; 17, EPS module; 18, transformation of acetate to pyr

The optimal status of Accumulibacter is maintained by the balance pair of carbon versus electrons in AN phase and the balance pair of electrons versus energy in AE phase. Based on the whole picture of Accumulibacter pan-pathway, we summarize nine scenarios to represent all optimal and suboptimal status (Figs. 3 and 4). Under optimal status (scenario A1), the primary sources of carbon, electron, and energy flows are balanced and Accumulibacter expresses the primary pathways (solid lines in Fig. 3). However, suboptimal status could impact the transcriptional patterns of Accumulibacter, as indicated by the other eight scenarios. We test this framework using two previously published metatranscriptional datasets (IMG/M-3300002341-3300002346 and NCBI-SRP038016) of clades IB and IIA [11, 41] from two acetate-feeding EBPR reactors (Additional file 1: Figures S3, S4 and Supplementary information). Even running under similar operational parameters, the reactor of clade IIA is stable and effective, while the reactor of clade IB experiences several deteriorations and has poor phosphorus removal performance.

#### The balance pair of carbon versus electrons

Accumulibacter can employ secondary modules to stabilize the slightly disturbed status in AN phase back to a balanced status for the following AE phase. When provided with excessive primary carbon source (acetate) than primary electron source (Gly) (scenarios B1–B3), Accumulibacter partitions acetate to provide additional electrons (no. 7 and no. 10 in Fig. 4; green-dotted lines in Fig. 3b1) and turns off the oxidative branch of split TCA cycle (no. 9 in Fig. 4) to reserve electrons. On the contrary, under the status of insufficient primary carbon source than primary electron source (scenarios C1–C3), Accumulibacter provokes modules that supplement additional carbon (no. 3–5 in Fig. 4; green-dotted lines in Fig. 3a1) and modules that consume electrons (no. 11 and no. 12 in Fig. 4; red-dotted lines in Fig. 3b1) to maintain redox condition.

Clade IB has overloading acetate (ccenario B1–B3), and Clade IIA has insufficient acetate (scenario C1–C3). In AN phase, carbon is flowing from acetate to PHA and electrons are flowing from glycogen to PHA in both clades. Only clade IIA is found to recruit secondary carbon sources (LCFAs, AAs, and glutamate), indicating that acetate is limited for clade IIA but not for clade IB. Regarding the downstream of carbon flow, clade IB seems to use the complete TCA cycles to generate more electrons, which suggests that the carbon source may not be a limiting factor for clade IB. In addition, the electrons provided by Gly are insufficient in clade IB and overloading in clade IIA. Complete and partial TCA cycles are highly expressed only in clade IB to supply secondary electrons. Instead, clade IIA employs modules to consume excessive electrons, including split TCA cycle (oxidative branch), hydrogenases, and denitrification. All these observations imply that the primary carbon source is overloading for clade IB and insufficient for clade IIA.

Both two clades successfully coordinate the secondary modules to stabilize the disrupted status in AN phase back to the balanced status for the next AE phase. Clades IB and IIA share similar expression profiles of carbon and electron flows in AE phase. Electrons are even enough for hydrogenases and denitrification in both clades (nos. 11 and 12 in Fig. 4; red-dotted lines in Fig. 3b2). Since primary energy source is determined by the preferability and availability of electron acceptors [42], when oxygen is sufficient, the other electron acceptors are mainly used for redox balance. It indicates that the electrons are excessive and that these two clades have recovered to a balanced status.

#### The balance pair of electron versus energy

When provided with limited phosphorus, Accumulibacter can store more electrons in AE phase to compensate energy in the AN phase; scenarios A2, B2, and C2 describe the unbalanced status of overloading phosphorus. These three scenarios display an increased expression of the electron flow from primary electron source (complete TCA cycle) to primary energy source (ETP) in AE phase (no. 7 and no. 15 in Fig. 4), to supply extra energy for phosphorus uptake and storage. On the contrary, in scenarios A3, B3, and C3, the status of inadequate phosphorus can cause the low expression of primary energy consumer (PolyP) and phosphate transporters (no. 13 and no. 14 in Fig. 4). Since this directly influences the primary energy source (PolyP) in the coming AN phase, more electrons (i.e., Gly) will be used for energy compensation in AN phase. Thus, in the current AE phase, Accumulibacter can allocate more Acyl-CoA to Gly module (no. 6 and no. 10 in Fig. 4; red-dotted lines in Fig. 3c2) for the flowing AN phase [43].

Clade IB has limited phosphorus (scenario B3), and clade IIA has sufficient phosphorus (scenario C1 and C2). In AN phase, PolyP provides energy for both clades and emits part of the intracellular phosphorus [44, 45], while the phosphate transporters (*pst* and *pit*) are expressed only in clade IIA. The low expression of PolyP module and phosphate transporters suggests that clade IB is provided with limited phosphorus, when additional energy is provided by the high expression of ETP. In AE phase, the ETP coupling with complete TCA cycle is highly expressed in clade IIA to provide energy. Instead, in clade IB, when limited phosphorus is provided, we find that partial TCA cycle is provoked for additional glycogen replenishment to fuel ETP in the following AN phase [43].

#### Evaluation of status: good or bad for enrichment

We propose that scenarios A1 and A2, and C1 and C2 are advantageous for Accumulibacter enrichment, to provide overloading phosphorus and slightly limited acetate (Fig. 4). Accumulibacter could prefer the acetate balance (scenarios A1–A3) or acetate shortage (scenarios C1–C3) situations than the acetate overloading (scenarios B1–B3) situation. Those unconsumed acetates will promote the unwanted growth of its competitors, such as glycogen-accumulating organisms (GAOs). In contrast, phosphorus overloading (scenarios A2, B2, and C2) is beneficial for Accumulibacter to compete carbon, while limited phosphorus (scenarios A3, B3, and C3) weakens the ability of Accumulibacter as PAOs and turns it into GAOs [43].

We demonstrate one good example (clade IIA) and one bad example (clade IB) for Accumulibacter enrichment. Two enrichment systems display totally opposite scenarios and should use different strategies for optimization. Clade IIA is fed with limited acetate and insufficient phosphorus, while clade IB is fed with sufficient acetate and inadequate phosphorus. Compared to clade IIA, the status of clade IB is the least favorable for Accumulibacter, in line with our operational experiences of this reactor for 4 years.

### Accumulibacter diversity: contribution from non-core-genomes

The diversity within Accumulibacter [46–48] contributed by the non-core-genomes could provide strategies to enrich specific clades. Generally, the flexibility of Accumulibacter is mainly related to metabolism, cellular processes, and environmental information processing (Additional file 1: Figure S5), especially in membrane transport, signal transduction, and metabolisms of amino acids and carbohydrates. Specifically, as to carbon sources, type II has an additional feature to reclaim carbon from cysteine and serine while type I could only use the common carbon sources of SCFAs (Fig. 2). This suggests that cysteine and serine could be the selective substrates in future studies to enrich type II and suppress type I. For the electron sources, the catalase (*cat*) associated with partial TCA cycle is found in clades IIA, IIC, and IA to reoxidize electrons [49]. It implies the flexibility of these clades in maintaining redox balance, and that hydrogen peroxide could be a selective force for these clades. Denitrification could be an advantage of clades IIA, IIC, IIF, IA, IB, and IC to use nitrate and nitrite as TEAs in AN phase. Besides, all clades in type II and clade IB in type I have the potential of nitrogen fixation, while the regulatory and nitrogen stabilization genes (*nifW* and *nifZ*) are only harbored by type II.

Overall, Accumulibacter type II, especially clade IIA, encodes more diverse adaptations than type I, which could explain the observation of a wide distribution of clade IIA in different wastewater treatment plants (WWTPs) [18]. This observation also implicates the importance of clade IIA and points out the priority of clade IIA in future studies.

## Conclusion

In this study, we present a comprehensive mining method to decode the hidden traits combining genetic and transcriptional datasets, to guide the enrichment of not-yet-cultured populations. We focus on the whole picture of the involvement and cooperation of pathways in the carbon, electron, and energy flows. A new transcriptional analysis is designed to diagnose the status of not-yet-cultured populations in the experimental systems. By doing this, the genomic and transcriptomic data could be linked to the environmental conditions, which could indicate a potential strategy to optimize the enrichment systems. This method is tested on a group of functional microbes by in silico analysis, the Accumulibacter. We find that Accumulibacter can coordinate multiple pathways to stabilize the disrupted status back to balance. This method could help diagnose and provide traits for the enrichment and even isolation of not-yet-cultured populations. We would like to point out the limitation of this study that no experimental validation has yet been conducted to test this method.

## Additional files

Additional file 1:Supplementary information. **Table S1.** Relationship between the coverage, accuracy, FN, and FP of core-, dispensable-, and strain-specific genomes (FN1=FP2, FN2=FP3, FN3=$\max \left[\mathrm{P}\left(\overline{\mathrm{G}}\mathrm{i}\right)\right]$). **Table S2.** The estimated completeness, contamination, and accession number of 13 available Accumulibacter draft genomes. **Table S4.** The estimated FN and FP rates of core-, dispensable-, and strain-specific genomes with different cutoff from 1 to 13. **Figure S1.** The technical flow of this study. **Figure S2.** A density curve showing the distribution of the occurrence frequency of genes in the Accumulibacter pan-genome, determined by the integrated alignment results. **Figure S3.** The comparison of RNA expression of type I and type II Accumulibacter in anaerobic and aerobic phases. The abbreviations of modules and chemical components are the listed in Fig 2. **Figure S4.** The dynamic pattern of RNA expression of clade IIA highlighted in the constructed Accumulibacter pan-genome pathway. Abbreviations: AN, anaerobic phase; AE, aerobic phase. **Figure S5.** The distribution of KEGG function types (brite types) of all non-redundant genes/KOs in Accumulibacter pan-pathway (core-, dispensable-, and strain-specific pathways). (DOCX 1880 kb)

Additional file 2: **Table S3.** The raw reads and normalized metatranscriptomic data as RPKM, MRPKM, CRPKM, and LCRPKM for clades IB and IIA. (XLSX 2562 kb)

Additional file 3: **Table S5.** Material and energy flow (electron, energy, and carbon) of each module in anaerobic (AN) compared to aerobic (AE) phase of an EBPR biochemical cycle. Production, consumption of material, and reaction potential for both directions in one phase were highlighted in green, red, and yellow, respectively. The abbreviations of modules and chemical components are the listed in Fig. 2. (DOCX 19 kb)

Additional file 4: **Table S6.** Providers and consumers of electron, energy, and carbon in anaerobic (AN) and aerobic (AE) phases of an EBPR biochemical cycle. Primary consumers or providers were highlighted in bold. The abbreviations of modules and chemical components are the listed in Fig. 2. (DOCX 17 kb)

## Data Availability

All data generated or analyzed during this study are included in this published article and its supplementary information files. All self-written scripts used in this study are available on https://github.com/caozhichongchong/PAPP.

## Abbreviations

AA: Amino acid
Acyl-CoA: Acetyl-CoA
AE phase: Aerobic phase
AN phase: Anaerobic phase
EBPR: Enhanced biological phosphorus removal
EPS: Exopolysaccharide
ETC: Electron transport chain
fdH_2_: Ferredoxin
Gly: Glycolysis/gluconeogenesis
LCFA: Long-chain fatty acid
N: Nitrogen
P: Phosphorus
PHA: Polyhydroxyalkanoates
PL: Phospholipid
PolyP: Polyphosphorus
Pro: Propionate
Pro-CoA: Propionyl-CoA
S: Sulfur
TCA cycle: Glycolysis and tricarboxylic acid cycle

## References

[1] Handelsman J (2004). Metagenomics: application of genomics to uncultured microorganisms. Microbiol Mol Biol Rev.

[2] Tripp HJ, Kitner JB, Schwalbach MS, Dacey JW, Wilhelm LJ, Giovannoni SJ (2008). SAR11 marine bacteria require exogenous reduced sulphur for growth. Nature.

[3] Vernikos G, Medini D, Riley DR, Tettelin H (2015). Ten years of pan-genome analyses. Curr Opin Microbiol.

[4] Lapierre P, Gogarten JP (2009). Estimating the size of the bacterial pan-genome. Trends Genet.

[5] Tettelin H, Masignani V, Cieslewicz MJ, Donati C, Medini D, Ward NL, Angiuoli SV, Crabtree J, Jones AL, Durkin AS (2005). Genome analysis of multiple pathogenic isolates of Streptococcus agalactiae: implications for the microbial “pan-genome”. Proc Natl Acad Sci U S A.

[6] Stewart EJ (2012). Growing unculturable bacteria. J Bacteriol.

[7] Martin HG, Ivanova N, Kunin V, Warnecke F, Barry KW, McHardy AC, Yeates C, He S, Salamov AA, Szeto E (2006). Metagenomic analysis of two enhanced biological phosphorus removal (EBPR) sludge communities. Nat Biotechnol.

[8] Skennerton CT, Barr JJ, Slater FR, Bond PL, Tyson GW (2015). Expanding our view of genomic diversity in Candidatus Accumulibacter clades. Environ Microbiol.

[9] Albertsen M, McIlroy SJ, Stokholm-Bjerregaard M, Karst SM, Nielsen PH (2016). “Candidatus Propionivibrio aalborgensis”: a novel glycogen accumulating organism abundant in full-scale enhanced biological phosphorus removal plants. Front Microbiol.

[10] Flowers JJ, He S, Malfatti S, del Rio TG, Tringe SG, Hugenholtz P, McMahon KD (2013). Comparative genomics of two ‘Candidatus Accumulibacter’ clades performing biological phosphorus removal. ISME J.

[11] Mao Y, Yu K, Xia Y, Chao Y, Zhang T (2014). Genome reconstruction and gene expression of “Candidatus Accumulibacter phosphatis” Clade IB performing biological phosphorus removal. Environ Sci Technol.

[12] Mao Y, Wang Z, Li L, Jiang X, Zhang X, Ren H, Zhang T (2016). Exploring the shift in structure and function of microbial communities performing biological phosphorus removal. PloS one.

[13] Zhang T, Liu Y, Fang HHP (2005). Effect of pH change on the performance and microbial community of enhanced biological phosphate removal process. Biotechnol Bioeng.

[14] Lu H, Oehmen A, Virdis B, Keller J, Yuan Z (2006). Obtaining highly enriched cultures of Candidatus Accumulibacter phosphates through alternating carbon sources. Water Res.

[15] Lopez-Vazquez CM, Song YI, Hooijmans CM (2007). Short-term temperature effects on the anaerobic metabolism of glycogen accumulating organisms. Biotechnol Bioeng.

[16] Lanham A, Moita R, Lemos P, Reis M (2011). Long-term operation of a reactor enriched in Accumulibacter clade I DPAOs: performance with nitrate, nitrite and oxygen. Water Sci Technol.

[17] Flowers JJ, He S, Yilmaz S, Noguera DR, McMahon KD (2009). Denitrification capabilities of two biological phosphorus removal sludges dominated by different ‘Candidatus Accumulibacter’clades. Environ Microbiol Rep.

[18] Zhang AN, Mao Y, Zhang T (2016). Development of quantitative real-time pcr assays for different clades of “Candidatus Accumulibacter”. Sci Rep.

[19] He S, Kunin V, Haynes M, Martin HG, Ivanova N, Rohwer F, Hugenholtz P, McMahon KD (2010). Metatranscriptomic array analysis of ‘Candidatus Accumulibacter phosphatis’-enriched enhanced biological phosphorus removal sludge. Environ Microbiol.

[20] Xia Y, Wang Y, Wang Y, Chin FY, Zhang T (2016). Cellular adhesiveness and cellulolytic capacity in Anaerolineae revealed by omics-based genome interpretation. Biotechnol Biofuels.

[21] Xia Y, Wang Y, Fang HH, Jin T, Zhong H, Zhang T. Thermophilic microbial cellulose decomposition and methanogenesis pathways recharacterized by metatranscriptomic and metagenomic analysis. Sci Rep. 2014;4;6708. 10.1038/srep06708 PMC4204047 25330991

[22] Chubukov V, Gerosa L, Kochanowski K, Sauer U: Coordination of microbial metabolism. 2014, 12:327. 10.1038/nrmicro3238 24658329

[23] Tettelin H, Riley D, Cattuto C, Medini D (2008). Comparative genomics: the bacterial pan-genome. Curr Opin Microbiol.

[24] Mongodin EF, Casjens SR, Bruno JF, Xu Y, Drabek EF, Riley DR, Cantarel BL, Pagan PE, Hernandez YA, Vargas LC (2013). Inter-and intra-specific pan-genomes of Borrelia burgdorferi sensu lato: genome stability and adaptive radiation. BMC Genom.

[25] Ahmed A, Earl J, Retchless A, Hillier SL, Rabe LK, Cherpes TL, Powell E, Janto B, Eutsey R, Hiller NL (2012). Comparative genomic analyses of 17 clinical isolates of Gardnerella vaginalis provide evidence of multiple genetically isolated clades consistent with subspeciation into genovars. J Bacteriol.

[26] Kanehisa M, Sato Y, Kawashima M, Furumichi M, Tanabe M (2016). KEGG as a reference resource for gene and protein annotation. Nucleic Acids Res.

[27] Huerta-Cepas J, Szklarczyk D, Forslund K, Cook H, Heller D, Walter MC, Rattei T, Mende DR, Sunagawa S, Kuhn M (2016). eggNOG 4.5: a hierarchical orthology framework with improved functional annotations for eukaryotic, prokaryotic and viral sequences. Nucleic Acids Res.

[28] Galperin MY, Makarova KS, Wolf YI, Koonin EV (2015). Expanded microbial genome coverage and improved protein family annotation in the COG database. Nucleic Acids Res.

[29] Green J, Paget MS (2004). Bacterial redox sensors. Na Rev Microbiol.

[30] Pirt S (1965). The maintenance energy of bacteria in growing cultures. Proc R Soc Lond B Biol Sci.

[31] Hardie DG, Scott JW, Pan DA, Hudson ER (2003). Management of cellular energy by the AMP-activated protein kinase system. FEBS Lett.

[32] Shannon P, Markiel A, Ozier O, Baliga NS, Wang JT, Ramage D, Amin N, Schwikowski B, Ideker T (2003). Cytoscape: a software environment for integrated models of biomolecular interaction networks. Genome Res.

[33] Oyserman BO, Moya F, Lawson CE, Garcia AL, Vogt M, Heffernen M, Noguera DR, McMahon KD. Ancestral genome reconstruction identifies the evolutionary basis for trait acquisition in polyphosphate accumulating bacteria. ISME J. 2016;10(12):2931. 10.1038/ismej.2016.67 PMC5148189 27128993

[34] Hesselmann R, Von Rummell R, Resnick SM, Hany R, Zehnder A (2000). Anaerobic metabolism of bacteria performing enhanced biological phosphate removal. Water Res.

[35] Wilmes P, Andersson AF, Lefsrud MG, Wexler M, Shah M, Zhang B, Hettich RL, Bond PL, VerBerkmoes NC, Banfield JF (2008). Community proteogenomics highlights microbial strain-variant protein expression within activated sludge performing enhanced biological phosphorus removal. ISME J.

[36] Burow LC, Mabbett AN, Blackall LL (2008). Anaerobic glyoxylate cycle activity during simultaneous utilization of glycogen and acetate in uncultured Accumulibacter enriched in enhanced biological phosphorus removal communities. ISME J.

[37] Oehmen A, Lemos PC, Carvalho G, Yuan Z, Keller J, Blackall LL, Reis MA (2007). Advances in enhanced biological phosphorus removal: from micro to macro scale. Water Res.

[38] Seviour RJ, Mino T, Onuki M (2003). The microbiology of biological phosphorus removal in activated sludge systems. FEMS Microbiol Rev.

[39] He S, McMahon KD (2011). Microbiology of ‘Candidatus Accumulibacter’ in activated sludge. Microb Biotechnol.

[40] Lin X, Handley KM, Gilbert JA, Kostka JE. Metabolic potential of fatty acid oxidation and anaerobic respiration by abundant members of Thaumarchaeota and Thermoplasmata in deep anoxic peat. ISME J. 2015;9;2740. 10.1038/ismej.2015.77 PMC4817634 26000553

[41] Oyserman BO, Noguera DR, Del Rio TG, Tringe SG, McMahon KD. Metatranscriptomic insights on gene expression and regulatory controls in Candidatus Accumulibacter phosphatis. ISME J. 2015. 10.1038/ismej.2015.155 PMC4796919 26555245

[42] Unden G, Bongaerts J (1997). Alternative respiratory pathways of Escherichia coli: energetics and transcriptional regulation in response to electron acceptors. Biochim Biophys Acta.

[43] Zhou Y, Pijuan M, Zeng RJ, Lu H, Yuan Z (2008). Could polyphosphate-accumulating organisms (PAOs) be glycogen-accumulating organisms (GAOs)?. Water Res.

[44] Tykesson E, Blackall L, Kong Y, Nielsen PH, la Cour Jansen J (2006). Applicability of experience from laboratory reactors with biological phosphorus removal in full-scale plants. Water Sci Technol.

[45] Schönborn C, Bauer H-D, Röske I (2001). Stability of enhanced biological phosphorus removal and composition of polyphosphate granules. Water Res.

[46] Saad SA, Welles L, Abbas B, Lopez-Vazquez CM, van Loosdrecht MC, Brdjanovic D (2016). Denitrification of nitrate and nitrite by ‘Candidatus Accumulibacter phosphatis’ clade IC. Water Res.

[47] Oehmen A, Carvalho G, Freitas F, Reis MA (2010). Assessing the abundance and activity of denitrifying polyphosphate accumulating organisms through molecular and chemical techniques. Water Sci Technol.

[48] Wang X, Wen X, Criddle C, Yan H, Zhang Y, Ding K (2010). Bacterial community dynamics in two full-scale wastewater treatment systems with functional stability. J Appl Microbiol.

[49] He S, McMahon KD (2011). ‘Candidatus Accumulibacter’ gene expression in response to dynamic EBPR conditions. ISME J.

