# Supplementary material for: Mining traits for the enrichment and isolation of not-yet-cultured populations
Source: Microbiome. 2019 Jun 25;7:96. doi: 10.1186/s40168-019-0708-4 (PMC6593511; doi:10.1186/s40168-019-0708-4)
Supplement: Supplementary file 1 — Supplementary information. Table S1. Relationship between the coverage, accuracy, FN, and FP of core-, dispensable-, and strain-specific genomes (FN1=FP2, FN2=FP3, FN3=$\max \left[\mathrm{P}\left(\overline{\mathrm{G}}\mathrm{i}\right)\right]$maxPG¯i). Table S2. The estimated completeness, contamination, and accession number of 13 available Accumulibacter draft genomes. Table S4. The estimated FN and FP rates of core-, dispensable-, and strain-specific genomes with different cutoff from 1 to 13. Figure S1. The technical flow of this study. Figure S2. A density curve showing the distribution of the occurrence frequency of genes in the Accumulibacter pan-genome, determined by the integrated alignment results. Figure S3. The comparison of RNA expression of type I and type II Accumulibacter in anaerobic and aerobic phases. The abbreviations of modules and chemical components are the listed in Fig 2. Figure S4. The dynamic pattern of RNA expression of clade IIA highlighted in the constructed Accumulibacter pan-genome pathway. Abbreviations: AN, anaerobic phase; AE, aerobic phase. Figure S5. The distribution of KEGG function types (brite types) of all non-redundant genes/KOs in Accumulibacter pan-pathway (core-, dispensable-, and strain-specific pathways). (DOCX 1880 kb) [file 40168_2019_708_MOESM1_ESM.docx]

**Supplementary Information**

**Manuscript title:**

Mining Traits for the Enrichment and Isolation of Not-yet-cultured Populations

An Ni Zhang^1^, Yanping Mao^1, 2^, Yubo Wang^1^, Tong Zhang^1^*

^1^Environmental Biotechnology Laboratory, Department of Civil Engineering, The University of Hong Kong, Pokfulam Road, Hong Kong

^2^College of Chemistry and Environmental Engineering, Shenzhen University, Shenzhen 518060, P.R. China

*Corresponding author:

Address: Environmental Biotechnology Laboratory, Department of Civil Engineering, The University of Hong Kong, Pokfulam Road, Hong Kong

Phone: +852-2857 8551

Fax: +852-2859 8987

Email: [zhangt@hku.hk](mailto:zhangt@hku.hk)

**Supplementary Methods**

**Definition of the cutoff for core-genome**

Because of the incompleteness of draft genomes, the strict criteria for core-genome in complete genomes may result in low coverage of core genes (high false negative (FN) rate). Thus, we introduced a new definition of core-genome as a gene shared by the cutoff of *n* genomes in a collection of *N* genomes (*n* ≤ *N*). The cutoff value *n* was determined as the maximum genome number when the FN rates of pan-genome subdivision calculated with this cutoff were less than 1%. In this study, the FN rate of core-genome equals to the false positive rate (FP) of dispensable-genome, which describes the probability of one core gene to be considered as dispensable for its absence in at least *N*-*n*+1 genomes because of their incompleteness (contained in the incomplete part of these draft genomes). Meanwhile, the FN rate of dispensable-genome equals to the FP rate of strain-specific-genome, which represents the probability of a dispensable gene (originally harbored in *m* genomes, *m* from 2 to *n*-1) to be considered as a strain-specific gene when it is present in only one genome and missing in the other randomly selected *m*-1 genomes. In this way, both the coverage (100%-FN) of core- and dispensable- genomes and the accuracy (100%-FP) of dispensable- and strain-specific- genomes were maintained to be more than 99% with cutoff *n* (Table S1). The accuracy (100%-FP) of core-genome was difficult to be estimated; and the minimum accuracy of strain-specific-genome (100%-FN) could be represented by the maximum genome incompleteness.

*The FN rate* *of core-genome and the FP rate* *of dispensable-genome*

The probability of one originally existent gene to be observed as present in one draft genome (referred to as$P\left( \mathrm{Gi} \right)$) could be considered as the completeness of one genome. The probability of observing the absence of one originally existent gene in the same genome is $1-P\left( \mathrm{Gi} \right)$ (referred to as $P(\bar{G}i)$).

Because the completeness of each draft genome is mutually independent event, the probability of observing the presence of one originally existent gene in genomes A1, A2, A3, …, Am and its absence in B1, B2, B3, …, Bn genomes could be calculated by (E1).

$$P=P\left( A1\bigcap A2\bigcap A3\ldots\bigcap\mathrm{Am} \right)*P\left( \bar{B}1\bigcap\bar{B}2\bigcap\bar{B}3\ldots\bigcap\bar{B}n \right)==\prod_{i=1}^{m} P\left( \mathrm{Ai} \right)*\prod_{j=1}^{n} P\left( \bar{B}j \right).$$

(E1)

The FN rate of core-genome represents the probability of one core gene (originally existent in *N* genomes) to be considered as dispensable for its absence in at least ($N - n+1$) genomes (contained in the incomplete part of these genomes), which equals to 1 – the probability of discovering one core gene to be contained in at least n genomes (not in their incomplete part). In pan-genome analysis on complete genomes, the cutoff (C) of core-genome is set as *N*, which means that one gene should be shared by all strains/genomes to be considered as a core gene. Thus, the probability of observing one core gene under the cutoff *N* was (E2). The FN rate of core-genome under the cutoff of *N* could be estimated as $1-\prod_{i=1}^{N} P\left( \mathrm{Gi} \right)$, which was 72% for *Candidatus* Accumulibacter (Accumulibacter) in this study (Table S4). This means that one core gene has 72% possibility to be considered as dispensable when it is missed in one random genome. In other words, the coverage of core genome could be deduced as 28% (1-72%).

$$P\left( C=N \right)=P\left( G1\bigcap G2\bigcap G3\ldots\bigcap\mathrm{GN} \right)=P\left( \bigcap_{i=1}^{N} \mathrm{Gi} \right)=\prod_{i=1}^{N} P\left( \mathrm{Gi} \right).$$

(E2)

If the cutoff for a core gene (core-genome) was defined as *N*-1, which means that the absence of a core gene is allowed in one random genome, the probability of discovering one core gene to be present in at least *N*-1 genomes could be calculated as $P\left( C=N-1 \right)$ using (E3). The FN rate for *N*-1 as cutoff for core-genome could be calculated as $1- P\left( C=N-1 \right)$.

$$P\left( C=N-1 \right)= P\left( C=N \right)+P\left( C=N-1 for G1 \right)+P\left( C=N-1 for G2 \right)+\ldots+P\left( C=N-1 for GN \right)=\prod_{i=1}^{N} P\left( \mathrm{Gi} \right)+P(\bar{G}1)\prod_{i=2}^{N} P\left( \mathrm{Gi} \right)+P\left( G1 \right)P(\bar{G}2))\prod_{i=3}^{N} P\left( \mathrm{Gi} \right)+\ldots+P(\bar{G}N)\prod_{i=1}^{N-1} P\left( \mathrm{Gi} \right)$$

(E3)

The FN rate for cutoff *n* of core-genome ranging from the value *N* to 1 was sequentially calculated in the same way as $1- P\left( C=N-n \right)$ to determine the cutoff value of core-genome for Accumulibacter (Tables S1 and S3).

*The FN rate of dispensable-genome and the FP rate* *of strain-specific-genome*

$$P\left( C=n \right)=P\left( m=n-1 \right)+P\left( m=n-2 \right)+\ldots+P\left( m=1 \right)=\frac{\sum_{i=1}^{C_{N}^{n-1}} \sum_{j=1}^{n-1} P(m=n-1 for Gj in i combination genomes)}{C_{N}^{n-1}}+P\left( m=n-2 \right)+\ldots+P\left( m=1 \right)=\frac{\sum_{i=1}^{C_{N}^{n-1}} [\sum_{j=1}^{n-1} [P\left( \mathrm{Gj} \right)*\prod_{k=1}^{n-2} P\left( \bar{G}k \right)]]}{C_{N}^{n-1}}+P\left( m=n-2 \right)+\ldots+P\left( m=1 \right)$$

(E4)

The FN rate of dispensable-genome under cutoff *n* equals to the probability of a dispensable gene, originally owned by *m* (*m*<*n*) genomes, is considered as strain-specific because of its presence in only one genome *j* and missing from all the other *m*-1 genomes due to their incompleteness (contained in their incomplete part). For cutoff *n*, a collection of *m* (2<*m*<*n*) genomes is randomly selected from *N* genomes to consist of a combination set *i*. In combination *i*, one genome *j* is randomly selected as the owner of a present gene, while the other *m*-1 genomes in combination *i* all miss this gene (contained in their incomplete part). The FN of dispensable-genome for genome *j* in combination *i* can be calculated as $P\left( \mathrm{Gj} \right)*\prod_{k=1}^{m-1} P\left( \bar{G}k \right)$. The sums of the FN rate of all conditions in combination *i* are merged with other combinations (*m* from 2 to *n*-1) extracted from *N* genomes (totally $C_{N}^{m}$ genome sets). The total sum is divided by the total number of combination sets $C_{N}^{m}$ and added with of FN rate of cutoff 2 to cutoff *n*-1, which comprises the FN rate of dispensable-genome under the cutoff *n* (E4).

Here as demonstrated by Accumulibacter, the FP and FN rates of dispensable-genome were evaluated with the cutoff *n* ranging from *N*-1 to 1 (Tables S1 and S3).

*The FN rate of strain-specific-genome and the FP rate* *of core-genome*

The maximum FN rate of a strain-specific gene is estimated as the maximum incompleteness of the collection of genomes as $P=max[P\left( \bar{G}i \right)]$, which represents the highest possibility of a unique gene to be missed due to the genome incompleteness (contained in the incomplete part of that genome). Because that the incompleteness part of genomes is unknown and unpredictable, the FP rate of core-genome was unable to be calculated.

*Assumptions for calculation*

The FN and FP rates of draft genomes under different cutoff *n* were calculated with the following assumptions of i) the completeness of draft genomes is mutually independent event in terms of probability theory; ii) the judgment of the cutoff *n* is based only on the number of sharing genomes while the phylogenetic relationships have no influence on the result. For Accumulibacter, we introduced another criterion that a gene shared only within one Type could not be defined as a core gene (core-genome), that is, the core-genome should present in both two Types.

**Clustering orthologous and non-orthologous genes by sequence-based alignment**

Genes of all Accumulibacter genomes were filtered by criteria: (i) genes with ≤ 50 codon length were excluded; (ii) genes with 51-100 codons were excluded with no intact match within all pan-genome [1]. Genes were defined as homologs with a minimum alignment of 50% identity over 50% of locus length [2, 3] by either one of the following comparison methods in BLAST+ program [4]: (i) a protein search against all the predicted genes by BLASTP; (ii) a DNA search of the predicted genes against the complete genome DNA sequences by BLASTN; (iii) a translated protein (different from “protein search”) search of the genes against the complete genome DNA sequence by TBLASTN [2, 3]. A gene was considered as core if it was shared by at least the cutoff of *n* genomes (occurrence frequency of *n*). Otherwise, this gene was labeled as a dispensable (shared by more than one genomes) or strain-specific (unique to one genome). The distribution of occurrence frequency of all genes was summarized by integrating all alignment results [5] to subdivide the pan-genome by comparing to the cutoff *n*. A density curve (Fig S2) was constructed to demonstrate the distribution of gene occurrence frequency, which was used to generate the genome sampling curves. The constructed curves (Fig 1a, 1b and 1c) agreed with the pattern generated by genome-centered approaches [1-3, 6].

**Pan-genome construction and sampling curves fitting**

Pan-genome was defined as the collection of all non-redundant genes of all genomes of a taxonomy lineage by removing replicated/orthologous genes. With each addition of a genome into a genome set, the number of core genes, new/unique genes (strain-specific genes of the added genome) and the size of pan-genome were calculated based on the occurrence frequency of genes in the current genome set. The total number (TN) of the possible combinations of the core and new/unique gene measurement when adding the *m*^th^ genome into the (*m*-1) genomes can be estimated as (E5) [2]:

$$TN=13!/[\left( m-1 \right)!*\left( 12-m \right)!]$$

(E5)

The N_core_ and N_dis_ (the cutoff of core-genome and strain-specific-genome) were re-estimated for each combination of genome sets by their completeness using the same method described before. Here, N_core_ was maximum number to maintain a less than 1% FN rate for the core-genome while N_dis_ was maximum number to maintain a less than 1% FN rate for the dispensable-genome. A combination of *m* genomes was considered as effective to be further preceded to the measurement of core and new genes only if the N_core_ and N_uni_ of this genome set met the following requirement (E6):

$$m-Ncore+1 \geq N\mathrm{dis}$$

(E6)

As demonstrated by Accumulibacter, an exhausted sub-sampling method was applied here to simulate sequential inclusion of up to 13 currently available Accumulibacter genomes in all possible permutation of genomes. The size of core-, strain-specific- and pan- genomes were extrapolated by fitting three exponential decaying functions (E7-E9) against the number of included genomes [2, 5, 7-9]. The pan-genome size was predicted based on the parameters $Ks, \tau s, tg(\theta)$ derived from the strain-specific-genome model (E8). The variation of core gene number by adding different *n*^th^ genome was caused mainly by duplicated genes and paralogs [2].

For core-genome size, $Fc=Kc exp\left[ -\frac{n}{\tau c} \right]+ \Omega$. (E7)

For strain-specific-genome size, $Fs=Ks exp\left[ -\frac{n}{\tau s} \right]+ tg(\theta)$. (E8)

For pan-genome size, $P\left( n \right)=D+tg\left( \theta\right)\left[ n-1 \right]+ Ks\exp\left[ -\frac{2}{\tau s} \right]\frac{1-\exp\left[ -\frac{n-1}{\tau s} \right]}{1-\exp\left[ -\frac{1}{\tau s} \right]}$. (E9)

The free parameters $\mathrm{Kc}$ and $\mathrm{Ks}$ were the amplitudes of the exponential decay; $\tau c$ and $\tau s$ were the decay constants for $\mathrm{Fc}$ and $\mathrm{Fs}$ converging; and $\Omega$ and $tg(\theta)$ measured the size of the genomes for *n*→∞, where $\theta$ was the extrapolated angle for pan-genome size $P\left( n \right)$ growth with new available Accumulibacter genomes; n was the number of genomes taken into consideration and $D$ was the average gene number of Accumulibacter genomes. From three models, $\Omega$ could be deduced the plateau of core genome size predicted by available genomes and the $tg(\theta)$ was estimated as the number of new genes expected from each new genome.

**Functional gene annotation**

All non-redundant pan-genome genes were annotated by KEGG Database (2016-11-21 version) [10] and eggNOG 4.5 *Betaproteobacteria* hmm database [11] integrated with COG Database [12] for organic and inorganic ion transporters. The KO number was assigned to each Accumulibacter gene with the criteria of 80% identity, 0.8 hit length ratio and 1e-2 *e*-value. Each KO was parsed into metabolic and non-metabolic pathways to generate and visualize highlighted KEGG pathways by Cytoscape KEGGScape [13, 14].

**Metatranscriptomic data normalization to CRPKM (cellular relative gene expression)**

*Definition of CRPKM (cellular reads per kilo base per million)*

The CRPKM (cellular reads per kilo base per million) was defined as the absolute transcriptional activity of a gene within one cell of a phylogenetic clade, which was also represented as the cellular RPKM. It was calculated based on the MRPKM (metatranscriptomic RPKM) [15] times the cellular copy number of one gene (E10). The sensitive limit of CRPKM detection can also be calculated by equation (E11). The cellular copy number of each gene can be estimated by homology or KEGG redundancy/occurrence in the reference genome or the draft bin from metagenomic data [15-17]. In other words, the cellular copy number of one gene equals to its gene occurrence in its genome. In this study, we used the same criteria described previous section [2, 3] to identify the homologs by (i) a protein search against all the predicted genes against itself by blastp [4].

To compare the CRPKM of different samples, the DNA and RNA extraction efficiencies should be taken in to consideration. However, if the DNA and RNA extraction efficiencies are not available, the absolute CRPKM of each sample can be converted into expression ranks to be compared between samples.

$$\mathrm{CRPKM}ij (Cellular RPKM of gene i in sample j) = \frac{\mathrm{RPKM}ij}{C}=MRPKMij * Gi=\frac{\mathrm{RPKM}ij\_DNA*\eta j\_\mathrm{RNA}}{\mathrm{RPKM}ij\_RNA*\eta j\_\mathrm{DNA}}* Gi=\frac{\frac{Ni}{Li}*\frac{{10}^{9}}{\mathrm{TN}j}*\eta j\_\mathrm{RNA}}{\frac{N'i}{Li}*\frac{{10}^{9}}{\mathrm{TN}^{'}j}*\eta j\_\mathrm{DNA}}* Gi$$

(E10)

$$CRPKM0=\frac{\frac{1}{average(L)}*\frac{10^9}{average(TN)}}{C}=\frac{\mathrm{average}\left( \mathrm{TN}^{'} \right)*average\left( \eta j\_\mathrm{RNA} \right)}{average(TN)*average\left( \eta j\_\mathrm{DNA} \right)}*average(Gi)$$

(E11)

Where N*i* is the number of mRNA reads in metatranscriptomic data mapped to gene *i* (N’*i* for DNA reads in metagenomic data); $\eta$ in the extraction efficiency of one sample *j*; TN*j* is the total number of mRNA reads in metatranscriptomic data in sample *j* (TN’*j* for DNA reads in metagenomic data); L*i* is the length of gene *i* in reference genome or transcriptome data; C is the average cell number and G*i* is the average genomic copy number of gene *i*; CRPKM0 is sensitive limit of an expressed gene that can be detected.

*CRPKM transformation and differential gene expression*

The CRPKM results were transformed by logarithm by formula $LCRPKM=log2(CRPKM + 1)$. Genes were classified in three RNA expression levels (1-4, I-blue, 5-7, II-orange, 8-9, III-red) according to their LCRPKM and visualized in the Accumulibacter pan-pathway.

Since the RNA and DNA extraction efficiencies were unavailable for Clades IB and IIA [18, 19], the absolute value of CRPKM in samples (52 min in anaerobic phase and 51 min in aerobic phase) of Clade IIA and (60 min in anaerobic phase and 60 min in aerobic phase) of Clade IB was transferred into expression ranks by the standard: the first 10% of all genes as the highly expressed level III-red, the 10% to 50% as mediated expressed level II-orange, the last 50% as low expressed level I-blue. The expression ranks of Clades IB and IIA was visualized in Accumulibacter pan-pathway.

**Pipeline summary**

The Pan-genome and Pan-pathway Pipeline (PAPP) for pan-genome analysis could be downloaded from <https://github.com/caozhichongchong/PAPP>, which included the following packages

1. Pancut for cutoff definition, FN and FP calculation and pan-genome subdivision;
2. KEGGMaster for pan-genome annotation (assigning KO numbers) and free-optimizing KEGG pathways construction;
3. Panfit for genome sampling curves construction and fitting;
4. Panpath for visualizing the new Accumulibacter genomes and new Accumulibacter metatranscriptomic datasets against Accumulibacter reference pan-pathway, specifically for AN and AE phases. The Accumulibacter pan-pathway constructed in this study was also provided in the PAPP package as reference for future studies;
5. CRPKM for metatranscriptomic datasets normalization to cellular relative gene expression, CRPKM (cellular reads per kilo base per million).

**List of abbreviations.**

For metabolites, 1,3BPG: 1,3-Bisphospho-D-Glycerate; 2-H-3-O P: 2-Hydroxy-3-Oxobutyl Phosphate; 2-PG: 2-Phosphoglycolate, 2-Phospho-D-Glycerate; 3-PG: 3-Phosphoglycerate; Ac: Acetate; AcAc-CoA: Acetoacetyl-CoA; Acyl P: Acetyl Phosphate; Acyl-[Acp]: Acetyl-[Acyl-Carrier Protein]; Acyl-CoA: Acetyl-CoA; ADP-Glu: ADP-Glucose; Amy: Amylose; APS: Adenosine 5'-Phosphosulfate; As: Arsenical; Dcarbon: Dicarboxylate; E4P: D-Erythrose 4-Phosphate; Beta-F1,6P: D-Fructose 1,6-Bisphosphate, Beta-D-Fructose 1,6-Bisphosphate; D-MM-CoA: D-Methylmalonyl-CoA; D-NAD: Deamido-NAD; Rubp: D-Ribulose 1,5-Bisphosphate; Ru5P: D-Ribulose 5-Phosphate; Fru: Frucose; G3-P: Glyceraldehyde 3-Phosphate; Glu: Glucose; Gly: Glycogen; Gly Amy: Glycogen Amylose; Glyc: Glycine; Glyrl: Glycerolipid ; Glyrpa: Glycerophosphoric Acid; Glyrpl: Glycerophospholipid; GTP: Guanosine Triphosphate; Hex-CoA: Hexadecanoyl-CoA; Asp: L-Aspartate; Cys: Cysteine; Lev: Levoluse; Lhser: L-Homoserine; L-MM-CoA: L-Methylmalonyl-CoA; L-OS: Lipo-Oligosaccharide; LP: Lipoprotein; LPS: Lipopolysaccharide; Mal-[Acp]: Malonyl-[Acp]; Mal-CoA: Malonyl-CoA; MFS: 3-Phenylpropionic Acid Transporter; Mupep: Muropeptides; Nri: Nicotinate Ribonucleotide; NMN, Nicotinamide D-ribonucleotide; P: Phosphate; Pa: Phosphonate; Pan: Pantothenate; PAP: Adenosine 3',5'-Bisphosphate; PAPS: 3'-Phosphoadenosine 5'-Phosphosulfate; PEP, Phosphoenolpyruvate; PL: Phospholipid (Phosphatidylethanolamine); EPS: Exopolysaccharide/Peptidoglycan; PL: Phospholipid; PHB: Polyhydroxybutyrate, Poly-Beta-Hydroxybutyrate; PHV. poly-3-hydroxyvalerate; PH_2_MV, poly-beta-hydroxy-2-methylvalerate; Pro: Propionate; Pro P: Propionyl Phosphate; Pro-CoA: Propionyl-CoA; Put: Putrescine; Pyr: Pyruvate; R5P: Ribose 5-Phosphate; Ribo: Riboflavin; S1,7BP: Sedoheptulose 1,7-Bisphosphate; S7P: Sedoheptulose 7-Phosphate; S3-HB-CoA: (S)-3-Hydroxybutyryl-CoA; Ser: Serine; THF: Tetrahydrofolate; Thi: Thiamin; Thr: L-Threonine; TMP: Thiamine Phosphat; Tsulfate : Thiosulfate; UDP-Murnac: UDP-N-Acetylmuramic Acid;; Ufpglu: Udpglucose; Glu: Alpha-D-Glucose; Alpha-G6P: Alpha-D-Glucose 6-Phosphate; Ala: Beta-Alanine; Beta-F6P: Beta-D-Fructose 6-Phosphate; Xu5P, D-Xylulose 5-Phosphate; Hse, L-Homoserine; LCFA, Long Chain Fatty Acid; Beta-G6P, Beta-D-Glucose 6-Phosphate; Beta-F6P, Beta-D-Fructose 6-Phosphate; Alpha-G1P: Alpha-D-Glucose 1-Phosphate; CDPD, CDP-Diacylglycerol; PPA, Phosphatidate; fd, Ferredoxin; Sper, Spermidine; His, Histidine; Lys, Lysine.

For modules, Split TCA Cycle-Re: Reductive branch of Split TCA Cycle (right arm); Split TCA Cycle-Ox: Oxidative branch of Split TCA Cycle (left arm); Dis, Dissimilatory; AN ETC, Anaerobic electron transport chain; AE ETC, Aerobic electron transport chain; AN ETP, Anaerobic electron transport phosphorylation; AE ETP, Aerobic electron transport phosphorylation; H Module, Hydrogen Module; ED, Entner–Doudoroff; EMP, Embden–Meyerhof–Parnas; Glycolysis/Gluconeogenesis, Gly; three modes of TCA Cycle, Complete, Partial and Split TCA Cycles; Polyhydroxyalkanoates, PHA; Long Chain Fatty Acid, LCFA; Phospholipid, PL; Propionate, Pro; Exopolysaccharide, EPS; Amino Acid, AA; Polyphosphorus, PolyP.

**Supplementary Results**

**Cutoff of core-genome and Accumulibacter genomic sampling curves fitting**

The cutoff *n* was defined by the criteria of less than 0.01 of FN for both core-genome (FP for dispensable-genome) and dispensable-genome (FP for strain-specific-genome) in maintenance of the coverage and accuracy of pan-genome classification. The FN for core-genome with the cutoff of 13, 12, 11, 10 and 9 were estimated as 7.15E-01, 3.24E-01, 9.48E-02, 1.84E-02 and 2.40E-03 while the FN for dispensable-genome with the cutoff of 12, 11, 10 and 9 were estimated as 1.61E-13, 6.41E-12, 1.82E-10 and 4.01E-09 (Table S4). Thus the cutoff of Accumulibacter core-genome was defined as 9. It was expected to cover more than 99% of core-genome and almost 100% of dispensable-genome with 99% accuracy. The maximum FN of strain-specific-genome was estimated as the maximum genomic incompleteness, which was 0.23 (AccIIF_2) in this study. Thus, the minimum coverage of strain-specific genes was expected to be 77%.

To estimate the core and global gene repertoire of the current genomes, the genome sampling curves of Accumulibacter pan-genome were fitted by the exponential decaying functions against the number of genomes. The best hit vector ($Kc,\tau c and \Omega$) of core-genome size was (3000, 3.04, and 1761) with correlation r^2^ as 0.98. The best hit vector for ($Ks,\tau s and tg(\theta)$ of strain-specific-genome size was (1234, 4.05, and 258) with r^2^ of 1.00. The pan-genome size function was fitted with the parameters $Ks,\tau s and tg(\theta)$ with the r^2^ of 1.00. The D for pan-genome model represented the average gene number of Accumulibacter genomes, which was 3634.

**The metabolism framework of Accumulibacter pan-pathway for EBPR anaerobic (AN) and aerobic (AE) phases**

*Carbon flow*

| Box1 Carbon AN (Figure 3a1) |
| --- |
| The main carbon source of AN phase is the extracellular carbon such as acetate or propionate (Pro). The acetate and Pro are transported by cation symporter (*actP*) to be further transferred to Acyl-CoA and propionyl-CoA (Pro-CoA) through both high affinity (*acs*, *prpE*) and low affinity (*ackA*, *pta*) pathways. In addition, glycolysis/gluconeogenesis (Gly) may provide additional but not primary intracellular carbon source; and long chain fatty acids (LCFAs) and amino acids (AAs) were also reported as supplementary carbon sources in AN phase [20]. Other alternative carbon sources include glutamate (from N module to TCA Cycle) and carbon fixation (by Calvin Cycle), which could be triggered by carbon famine at AN ending.  The major carbon consumer is polyhydroxyalkanoates (PHA) module to form polyhydroxybutyrate (PHB, requiring Acyl-CoA) and poly-3-hydroxyvalerate (PHV, requiring Acyl-CoA and Pro-CoA) [21]; while the alternative modules, including Complete TCA Cycle, Phospholipid (PL) module and LCFA module, are minor carbon consumers. It was notable that, in contrast to Split and Partial TCA Cycles, the Complete TCA Cycle may be employed to supply reducing power at the cost of Acyl-CoA.  In AN phase, some intermediate modules with no carbon consumption also exhibit essential function of carbon transformation, which include the Split TCA Cycle and Partial TCA Cycle. The oxidative branch (left arm) and reductive branch (right arm) of Split TCA Cycle [22] together with Partial TCA Cycle could convert Acyl-CoA to Pro-CoA bypassing from succinyl-CoA to generate PHV [23, 24]. |

The main carbon source of AN phase is the extracellular carbon such as acetate or Pro. Acetate and Pro are transported by cation symporter (*actP*) and then transferred to Acyl-CoA and Pro-CoA respectively through both high affinity acetyl-CoA synthetase (*acs*) or propionyl-CoA synthetase (*prpE*) and low affinity (*ackA*) acetate kinase together with (*pta*) phosphotransacetylase. Gly of Embden–Meyerhof–Parnas (EMP) may provide intracellular carbon sources together with the transformation of pyruvate (Pyr) to Acyl-CoA. LCFAs and AAs were also reported to be recruited as carbon sources in AN phase [20]. The net increase of fatty acid such as palmitate was detected during AN phase [19, 25, 26]. Although the LCFA was proposed to be converted to phospholipid (PL) to form encapsulate intracellular PHA granules and cell membrane [25, 27], they are two identical modules parting from the carbon hub Acyl-CoA and PL module could route carbon from multiple sources other than LCFA. Accumulibacter might utilize LCFA as carbon and electron sources [28] under AN carbon famine condition, instead of PL synthesis. Besides, extracellular and intracellular glutamate could also serve as a carbon provider to enter TCA Cycle through *gltIJKL* ABC transporter and *gltBD* glutamate synthase. Other alternative carbon sources include carbon fixation by Calvin Cycle, which could be triggered by carbon famine at AN ending. The pentose pathway from glycogen, glucose (Glu) also share partial pathways with Gly and Calvin Cycle, whereas it is incomplete in Accumulibacter pan-pathway to transfer Glu to D-ribulose 5-phosphate (Ru5P).

The major consumer of Acyl-CoA and Pro-CoA is PHA module to form PHB (requiring Acyl-CoA), PHV (requiring both Acyl-CoA and Pro-CoA) and poly-beta-hydroxy-2-methylvalerate (PH2MV, requiring Pro-CoA) [21]. Alternative modules including Complete and TCA Cycle, PL and LCFA modules are minor consumers of carbon. In contrast to Split and Partial TCA Cycles, the Complete TCA Cycle may be employed to supply reducing power at the cost of Acyl-CoA.

In AN phase, some intermediate modules with no consumption also exhibit essential function of carbon transformation, which include the Split TCA Cycle and Partial TCA Cycle. The oxidative branch (left arm) and reductive branch (right arm) of Split TCA Cycle together with Partial TCA Cycle could convert Acyl-CoA to Pro-CoA bypassing from succinyl-CoA to generate PHV [23, 24]. The production (averagely 10%) of PHV in acetate and Pro switching and Ac-feeding EBPR reactors [23, 24] was supposed to provide the evidence for Split TCA expression and the leaving of succinyl-CoA from TCA Cycle to produce Pro-CoA. It was also supported by the activity expression of corresponding genes, methylmalonyl-CoA mutase (*mut*) to transfer succinyl-CoA into L-methylmalonyl-CoA (L-MM-CoA) and methylmalonyl-CoA decarboxylase (*mmcD*) for D-methylmalonyl-CoA (D-MM-CoA) to Pro-CoA [19]. However, this is inconsistent with the missing gene *mcee* epimerase to convert L-MM-CoA to D-MM-CoA in Accumulibacter pan-genome. A possible assumption is that Accumulibacter could have some unknown epimerase for converting L-MM-CoA to D-MM-CoA to complete this pathway, or another unknown *prpB* gene (methylisocitrate lyase) to transfer succinate to Pro-CoA through methylisocitrate. The decrease of cellular relative expression level between *mut* and *mmcD* may suggest the interconversion between L-MM-CoA to D-MM-CoA could be the limiting step in Pro module.

| Box2 Carbon AE (Figure 3a2) |
| --- |
| In AE phase, the primary carbon pool is contributed by the PHA produced in the previous AN phase. The dissimilarity between the PHA and Gly modules is that the PHA serves as the primary carbon pool in AE phase; while in AN phase, acetate and Pro are the primary carbon sources and Gly is employed preferentially as an electron pool. Besides, Calvin Cycle could also supply additional carbon, but it was predicted functional mainly in anoxic condition [21]. In the meanwhile, the remaining acetate and Pro are routed towards Acyl-CoA and Pro-CoA conversion (entering the TCA Cycle through Pro module). Even though the TCA Cycle driven by Acyl-CoA yields more electrons than by Pro-CoA, the interconversion between Acyl-CoA and Pro-CoA is limited by the exhausted free acetate during the AE phase.  Complete TCA Cycle is the major consumer of carbon sources in AE phase, by fully oxidizing Acyl-CoA to generate ATP. In terms of electron and energy production, Partial TCA Cycle could be an unfavorable approach to partition Acyl-CoA. However, Partial TCA Cycle is an intermediate module in carbon flow to transform Acyl-CoA to oxaloacetate. By doing so, the Accumulibacter is able to control the amount of carbon (Acyl-CoA) for Gly module by shunting the decarboxylation steps of Complete TCA Cycle [23]. Additionally, LCFA, PL, AA, N (glutamate/glutamine) and exopolysaccharide (EPS) modules utilize the carbon sources mainly for cell component synthesis in AE phase. |

In AE phase, the primary carbon pool is contributed by the PHA granules produced at the previous AN phase. The dissimilarity between PHA and Gly carbon pools is that the PHA serves as the primary carbon pool in the whole AE famine phase, while in AN carbon feast phase, extracellular acetate and Pro contribute to the primary carbon source and Gly module is employed preferentially as an electron pool for Accumulibacter than carbon pool. Calvin Cycle could also supply additional carbon by carbon fixation; while it was predicted that Accumulibacter could only fix carbon in anoxic condition because only the large chain *rbcL* of the crucial rubisco gene was discovered in Accumulibacter pan-genome [21]. Thus, the Calvin Cycle was more likely to be activated at the AN phase ending and the AE phase beginning. However, the activity of Calvin Cycle was observed to be increasing at the end of AE phase [19], which could be more beneficial for Accumulibacter to provide additional carbon source during intracellular carbon famine period. Further experiments are necessary to confirm the potential of carbon fixation in AE carbon famine conditions. In the meanwhile, the remaining extracellular acetate and Pro are routed towards Acyl-CoA and Pro-CoA conversion. The products from the putative carbon pools are transformed to Acyl-CoA and Pro-CoA to be further allocated into multiple modules for energy generation and cell component synthesis. The Pro-CoA may enter the TCA Cycle by D-MM-CoA through Pro module or be converted directly to Acyl-CoA by transferase gene (*pct*) in Accumulibacter pan-pathway. Even though the TCA Cycle driven by Acyl-CoA may yield more electrons than Pro-CoA, the interconversion of Acyl-CoA and Pro-CoA is supposed to be limited by the exhausted acetate and Pro at AE phase of a typical EBPR process.

Complete TCA Cycle fully oxidizes Acyl-CoA into CO_2_ to generate ATP coupling with AE ETC, as the major consumer of carbon sources in AE phase. Due to the production of fewer electrons and no energy compared to Complete TCA Cycle, Partial TCA Cycle could be a relatively unfavorable approach to partition Acyl-CoA. However, Partial TCA Cycle is an intermediate module in carbon flow to transform Acyl-CoA to oxaloacetate. By doing so, it was able to control the amount of carbon (Acyl-CoA) for Gly module by shunting the decarboxylation steps of Complete TCA Cycle [23].The activity of Partial TCA Cycle in AE phase was also confirmed in several studies [22, 23]. Thus, the Acyl-CoA could be competed by both Complete and Partial TCA Cycle in AE phase for different purposes. In addition to TCA Cycle, the Gly module also requires Acyl-CoA to generate glycogen. LCFA, PL, AA, N (glutamate/glutamine) and EPS modules utilize carbon to synthesis cell components for cell growth and reproduction.

Two component systems also participate in the regulation of carbon flow. Accumulibacter may employ succinate transporter *dctA* in response to extracellular succinate. In AE phase with less N_2_ availability, *glnBDGL* genes may positively regulate the N module to transfer 2-oxoglutarate from TCA Cycle to synthesize glutamate or glutamine for additional nitrogen source.

*Electron flow*

The carbon flow is usually accompanied and motivated by electron transporting.

| Box3 Electron AN (Fig 3b1) |
| --- |
| The electrons (reducing agents/equivalents) are primarily contributed by Gly module in AN phase. Moreover, Complete, Partial and Split TCA (reductive branch) Cycles, N (glutamine/glutamate) and LCFA modules could serve as alternative but secondary providers.  In AN phase, the major consumer of electrons is PHA synthesis [29, 30]. Like most assimilatory metabolism, electrons in the form of NADPH are required in assimilatory pathways such as PHA module and Calvin Cycle [19, 21], while the electrons available are mainly in the form of NADH, fdH_2_, FADH_2_ and QH_2_ excluding a few NADPH generated directly by LCFA module. Thus, it is crucial for Accumulibacter to maintain the balance between the transformation (transhydrogenases) and the generation of reducing agents, which may affect the direction of modules to maintain redox condition. Such modules include TCA Cycles (Complete, Partial and the oxidative branch of Split TCA Cycles as providers and the reductive branch of Split TCA Cycle as a consumer) and LCFA module. In addition, the N module (dissimilatory nitrogen reduction and denitrification), S module, AN ETP and hydrogenases could also consume the excessive electrons. In Accumulibacter, AN ETP was proposed possible with cytochrome *b/b6* oxidase [21] using nitrate, nitrite and fumarate as terminal electron acceptors (TEAs) [23, 28, 31]. Thus, during a typical EBPR cycle when Accumulibacter has enough glycogen storage, these flexible modules could serve as electron consumers instead of carbon providers, especially at AN ending, to maintain the recycle of electron carriers. |

In AN phase, the electrons (reducing agents/equivalents) were primarily contributed by Gly from glycogen to Pyr and subsequently from Pyr to Acyl-CoA. Complete, Partial and Split TCA (reductive branch) Cycles, N (glutamine/glutamate) and LCFA modules could serve as alternative but secondary providers of additional reducing power. It was reported that almost 25% of reducing power for PHA module was produced by the TCA Cycle in AN phase [24] consuming 4% Acyl-CoA. Even though the glyoxylate shunt of Partial TCA Cycle and the reductive branch of Split TCA Cycle yield only 25% [24] and 50% of the reducing power that can be produced by Complete TCA Cycle, their independence on ETP and their function of transforming Acyl-CoA for PHA module (more PHV and less PHB) could be considered as their advantages especially in the AN phase. When the reducing power was forced to be balanced, Complete TCA Cycle could be activated at the cost of carbon (Acyl-CoA).

The major consumer of electrons is PHA synthesis [29, 30]. Like most assimilatory metabolism, the reducing agent in the form of NADPH is required in PHA module and Calvin Cycle [19, 21], while the reducing agents available are mainly NADH, fdH_2_ (Pyr to Acyl-CoA), FADH_2_ (Complete TCA Cycle and LCFA) and QH_2_ (Complete TCA Cycle) excluding a few NADPH directly generated by LCFA module. Thus, it is crucial for Accumulibacter to maintain the activity and efficiency of both the transformation of reducing agents (transhydrogenases) and the generation of primary reducing agents. In addition, the speed of NADH to NADPH transformation compared to the speed of NADH generation may affect the direction of module pathways to maintain the redox balance. Such models include the three modes of TCA Cycle (Complete, Partial and the reductive branch of Split TCA as providers and the oxidative branch of Split TCA as a consumer) and LCFA (provider and consumer) as flexible modules that could be driven by the redox condition, which has been supported by several studies. For example, by passing through Split TCA Cycle (oxidative branch), PHV was proposed to be produced instead of PHB forced by excessive reducing power [24]. The net production of LCFA in AN phase reported by Wexler and colleagues [25] may suggest the potential of LCFA module to balance cell redox state. Moreover, the N module (dissimilatory nitrogen reduction and denitrification), S module (sulfite to H_2_S), AN ETP and hydrogen gas (H_2_) production by hydrogenases [19] could also use excessive electrons. Accumulibacter pan-genome only has assimilatory S reduction pathways, which limits the potential of Accumulibacter to use sulfate directly to maintain cell redox homeostasis. However, the active expression of sulfate to adenosine 5'-phosphosulfate (APS) and 3'-phosphoadenosine 5'-phosphosulfate (PAPS) to sulfite indicated that Accumulibacter may possess unknown enzyme to convert APS to PAPS [19]. Accumulibacter could also use sulfite reductase (*cysI*) to re-oxidize NADPH. The AN ETP was proposed to be possible with cytochrome *b/b6* oxidase (*cyb/b6*) predicted in Clade IIA complete genome AccIIA_1 [21] using nitrate, nitrite and fumarate as terminal electron acceptors (TEAs) [23, 28, 31]. In ETP, the reducing power of NADH is firstly transferred to QH_2_ by Complex I (C.I, NADH dehydrogenase) and Complex II (C.II, succinate dehydrogenase) and then to TEA by Complex III (C.III, *cyb/b6*). Since the redox potential of fumarate/succinate redox pair is 0.03 in standard situation compared to 0.045 for Q/QH_2_, the accumulation of QH_2_ is critical to trigger this reaction. During a typical EBPR process when Accumulibacter has enough glycogen storage, these flexible modules could serve as electron consumers instead of carbon providers, especially at AN ending, to maintain the recycle of electron carriers.

| Box4 Electron AE (Fig 3b2) |
| --- |
| The major electron donor in AE phase is Complete TCA Cycle (NADH, FADH_2_, QH_2_); and PHA module and Partial TCA Cycle may also provide additional electrons in the form of NADH.  In AE phase, the reduced agents are mainly used by ETP (NADH, QH_2_) for energy generation and Gly module (NADH) for glycogen production. Unlike in AN phase that Complete TCA Cycle is limited by the availability of TEAs, with abundant oxygen in AE phase, Complete TCA Cycle would be more favorable compared to Partial TCA Cycle in terms of electron and energy generation. However, as discussed before, Accumulibacter could utilize the Partial TCA Cycle to control the flow from Acyl-CoA for glycogen replenishment. The flexible coordination between Partial and Complete TCA Cycles to control the carbon, electron and energy flows is a crucial ecological benefit endorsed by Accumulibacter to diverse niches. Besides, the LCFA, PL, EPS and Calvin Cycle modules also consume electrons in the form of NADH and NADPH for synthesis and carbon fixation. |

In AE phase, the major electron donor is supplied by Complete TCA Cycle (NADH, FADH_2_, QH_2_). PHA module and Partial TCA Cycle may also provide additional reducing power in the form of NADH.

The reduced coenzymes are mainly used by ETP (NADH, QH_2_) for further energy generation and Gly module (NADH) of Gly module, following the transformation of Acyl-CoA to Pyr (NADH, fdH_2_). Unlike in AN phase that Complete TCA Cycle could be limited by the redox potential and availability of TEAs, with abundant oxygen in AE phase, Complete TCA Cycle would be more favorable for Accumulibacter compared to Partial TCA Cycle in terms of obtaining more reducing power and energy. However, as discussed before, Accumulibacter could utilize the Partial TCA Cycle to control the carbon flow from Acyl-CoA for Gly module, bypassing the Complete TCA Cycle. The flexible coordination between Partial and Complete TCA Cycle to control the carbon, electron and energy flows is a critical ecological benefit endorsed by Accumulibacter to diverse niches with dynamic carbon sources and redox condition. The LCFA, PL, EPS and Calvin Cycle modules also consume electrons in the form of NADH and NADPH for synthesis and carbon fixation.

*Energy flow*

| Box5 Energy AN (Fig 3c1) |
| --- |
| The major energy provider in AN phase is polyphosphorus (PolyP); while Gly module and Complete TCA Cycle may supply additional but minor energy. Proton motive force (PMF) is required to generate ATP by F-type and P-type ATPases in ETP, which is created by Complexes I and III or accumulated previously by proton pumps.  The energy was used by the activation of acetate and Pro to Acyl-CoA and Pro-CoA plus ABC transporters. Additionally, the proton (*pntA/B*) and Na^+^ (*hppA*) pumps consume the ATP and pyrophosphate (*PPi*) to change the PMF for transporters; meanwhile the N module, LCFA module, S module, Split TCA Cycle and Calvin Cycle also consume ATP by substrate-level phosphorylation. |

Accumulibacter stores energy in the form of PolyP in the AE phase and uses PolyP as the primary energy source for in the AN phase. Gly module (ATP and GTP) and Complete TCA Cycle (GTP) coupling AN ETP may also provide additional energy. In a typical EBPR process, the upregulation of Complete TCA Cycle is possibly triggered by redox condition, rather than energy famine. PMF is required to generate ATP by F-type and P-type ATPase in ETP, which is created by C.I. and C.III Complexes and accumulated previously by proton pumps.

In AN phase, the energy was used by the transformation of acetate and Pro to Acyl-CoA and Pro-CoA plus the ABC transporters. Proton (*pntA/B*) and Na^+^ (*hppA*) pumps consume the ATP and pyrophosphate (*PPi*) to change the PMF for ATPase, electrochemical potential driving transporters and pores ion channels. The *PPi* is mainly produced by high affinity *acs* and PolyP depolymerization. The N module, LCFA module, S module, Split TCA Cycle and Calvin Cycle also consume ATP while mainly in the form of substrate-level phosphorylation.

| Box6 Energy AE (Fig 3c2) |
| --- |
| The primary energy pool in AE phase is Complete TCA Cycle coupling with AE ETP.  The generated energy is mainly consumed and stored in PolyP while Gly module also requires GTP and ATP for glycogen generation. In addition, the Pro module consumes ATP to enter the TCA Cycle. Another approach for Pro utilization is the direct transformation of Pro-CoA to Acyl-CoA, which could be more preferable for bonus electron production and no energy consumption, while it is limited by the available acetate during the AE phase. Other potential consumers of energy are LCFA, EPS, S (assimilatory S reduction), Calvin Cycle and N modules (mainly by nitrogen fixation). |

The energy pool in AE phase is Complete TCA Cycle (in the form of GTP) coupling with AE ETP (in the form of ATP) using oxygen as TEAs.

The generated energy is mainly consumed and stored in PolyP. Gluconeogenesis of Gly module also requires GTP and ATP to generate glycogen. In addition, the conversion of Pro-CoA to D-MM-CoA requires ATP to enter the TCA Cycle. Another approach is the direct transformation of Pro-CoA to Acyl-CoA by *pct*, which could be more preferable for bonus electron production and no energy consumption for Acyl-CoA to enter the TCA Cycle, while it is limited by the available acetate as discussed before. Other potential consumers in AE phase are LCFA, EPS, S (assimilatory S reduction) modules and Calvin Cycle.

Even though the interconversion of glutamate and glutamine is coupled by the conversion of ATP and ADP, its carbon flow is more likely to be dominated by the mutual transformation of glutamate/glutamine to 2-oxoglutarate from TCA Cycle in both two phases. Thus, the energy consumption in N module is mainly contributed by nitrogen fixation, as an energy expensive process.

**Expressional patterns of carbon, electron and energy flows in Accumulibacter pan-pathway during a typical EBPR cycle**

*AN Carbon flow*

The extracellular acetate and Pro were actively transported by *actP* by both Clades IB (level II) and IIA (level III). Accumulibacter was able to transfer the acetate into the carbon hub Acyl-CoA through both high and low affinity pathways before further consumption, while the low affinity pathways *ackA* with *pta* seemed to be active only in Clade IIA (level II-III). These findings suggested that Clade IIA might be more competitive in transporting and transforming acetate, while for Clade IB, transformation acetyl phosphate (Acyl P) to Acyl-CoA by *pta* could be a limiting step in terms of acetate or Pro uptake. Another possible explanation was that Clade IIA was fed with insufficient acetate source since the activity of *actP* was associated with low concentration of acetate [21, 32]. As stated before, it was inferred only based on the relative transcriptional level, which required further validation.

It was noticeable that Pro module of Clade IIA was employed. The generation of Pro-CoA to further synthesize PHV was also reported in other acetate-feeding reactor [24]. The conversion from Pro to Pro-CoA shared the same genes with acetate to Acyl-CoA, except for *prpE*. Thus, even with high expression (level II-III), the extracellular Pro was less likely to be the main source for these acetate-feeding reactors. Moreover, the inaccessibility of Pro for *pct* may rule out the contribution directly from Acyl-CoA, resulting in the succinyl-CoA from Split TCA Cycle as the main source of Pro-CoA in this case. The oxidative branch of Split TCA Cycle was also highly expressed at level III in Clade IIA, especially the fumarate reductase (*frdB*) and succinyl-CoA synthetase (*sucCD*) before entering the Pro module. The decreased expression of oxidoreductase *korAB* (level II) to convert between succinate-CoA and 2-oxoglutarate indicated that the carbon flow was shunted into Pro module instead of continuously flowing inside TCA Cycle. Together with the week activity of the glyoxylate shunt, it could be inferred that the majority of succinyl-CoA was contributed by the oxidative Split TCA Cycle. In contrast, no evidence was identified for Clade IB to convert Pro-CoA through succinyl-CoA by methylmalonyl-CoA mutase (*mut*) implying a week expression of Split TCA Cycle. In the meanwhile, it also indicated that the high expression of TCA Cycle (level II-III) was mainly contributed by Complete and Partial TCA Cycles in the forward TCA direction for Clade IB instead of the oxidative branch of Split TCA Cycle in the reverse TCA direction for Clade IIA. In addition, the expression of Partial TCA Cycle (forward direction) of Clade IB (level II) was more activated than Clade IIA (level I-II), while flowing to oxaloacetate instead of Pro-CoA. The direction of TCA Cycles was likely to be affected by cell redox condition rather than carbon source availability, which was further discussed below in the section of electron flow. Further investigation into the ability of Clade IB to active Pro-CoA and generate PHV must be conducted in Pro-feeding EBPR-reactors to confirm this potential.

Besides the extracellular acetate and Pro, Gly module could provide extra intracellular carbon source, which was highly expressed in both Types (level III). In the previous cycle, Accumulibacter stored glycogen instead of fructose (Fru) and Glu since the carbon flow from the latter two sources was limited (level I-II). The Gly module was likely to be limited by phosphoglycerate kinase (*pgk*) in both Types for transforming 1,3-bisphospho-glycerate (1,3BPG) to 3-phosphoglycerate (3-PG).

It was noticeable that Clade IIA could transport and recruit alternative carbon sources, such as glutamate/glutamine (level II-III) and AAs (II) into TCA Cycle or 3-PG, while Clade IB showed none or negligible expression of related modules and regulators. Clade IIA also exhibited more activity to route carbon from LCFA than Clade IB. Besides, Calvin Cycle was slightly activated (level I-II) to fix carbon for two Clades. It could be limited mainly by the first and last steps, the rubisco and the phosphoribulokinase (*prkB*). These observations suggested that Clade IIA tended requisition more diverse carbon sources than Clade IB. The assumption that Clade IIA might suffered from limited extracellular carbon sources could serve as a possible explanation.

The carbon in AN phase was mainly consumed by PHA module, which was both highly activated at level III in two Types. The Complete and Partial TCA Cycle partitioned the carbon flow from Acyl-CoA to balance redox status in Clade IB (level II-III). Other supplementary carbon consumers, such as PL, were detected at low expression level (0-I), which implied almost none additional carbon consumption. Accumulibacter might allocate available PL for PHA granules formation instead of direct synthesis in AN phase.

*AN Electron flow*

Gly module together with the conversion of Pyr to Acyl-CoA (level III) were considered as the major electron pool (NADH and fdH_2_) for Accumulibacter, with continuously active expression in both Types to generate reducing power (Fig S3). Additional potential electron donors from Complete and Partial TCA Cycle (providing NADH, QH_2_ and FADH_2_) were only highly expressed in Clade IB (II-III). N module was only actively expressed in Clade IIA (glutamate/glutamine) to generate NADH or NADPH, while its major contribution would be supplying more carbon budgets into TCA Cycle.

The PHA module of Accumulibacter, as both the carbon and electron (NADPH) sinks, was increasing activated (level III) for two Types. It was indicated that more alternative electron acceptors were motivated by Clade IIA, judging from the active expression of the oxidative branch of Split TCA Cycle (NADH, FADH_2_ and QH_2_), sulfite reduction (NADPH), dissimilatory nitrite reduction and denitrification (NADH and NADPH). Even though the ability of Accumulibacter to utilize sulfite and sulfate as electron acceptors may be affected by assimilatory S reduction, the activity of S module could partially imply the cell redox condition. In addition, the saline influence for IB reactor should provide more available S containing components for S module.

In AN ETP (NADH and QH_2_), the expression of fumarate (level III), the nitrate (level III) and nitrite reductases (level II-III) in both Clades demonstrated the ability of Accumulibacter to use nitrate, nitrite and fumarate as TEAs in AN phase. Moreover, the nitrate reduction was mainly contributed by denitrification (*napA*) for the low expression level 0-I of both the *narGHI* and assimilatory reductase *nasA*. However, as stated before that *napA* was missing in other Clades of Type II, the ability of denitrification by Clade IIA may not be representative enough for other Clades in Type II. The decreased expression of nitrite, nitric oxide and nitrous-oxide reductases in two Clades may suggest that Accumulibacter operated partial denitrification for ETP. The activity of hydrogenases (*hoxFHUY*) was also detected in two Types (level II-III) to release reducing power of NAD(P)H to H_2_.

Overall, Clade IB seemed to employ more electron-producing and less electron-consuming modules to provide reducing power than Clade IIA. Complete TCA Cycle of Clade IB was even activated in addition to Partial TCA Cycle to produce extra reducing power at the cost of carbon sources while Clade IB tended to recruit no supplementary carbon budgets. Based on these observations, we hypothesized that Gly and Partial TCA Cycle may not provide enough reducing power and extracellular acetate and Pro could provide adequate carbon sources for Clade IB. On the contrary, Clade IIA could be supplied with limited extracellular carbon sources and excessive reducing power for PHA synthesis. These differences could be interactively resulted from both environmental parameters and regulation abilities.

Since the majority of reducing equivalents require transformation to be directly used by electron-consuming modules, the activity and efficiency of transhydrogenases are essential for Accumulibacter. For example, the expression of fd-NADP reductase (*fpr*) in two Types was highly expressed (level II-III) consistently with the transformation of Pyr to Acyl-CoA (level III) to convert the fdH_2_ for further usage. The interconversion between NADPH and NADH by NAD(P) transhydrogenase (*pntAB*) was also highly activated at level II-III. It was proposed that the transformation of NADH to NADPH was slower than the generation of NADH, which could serve as another cause of the accumulation of NADH in Clade IIA [19], which further provoked the activation of multiple electron-consuming modules in Clade IIA.

*AN Energy flow*

The depolymerization of PolyP to provide ATP and GTP was highly expressed in both Types (level II-III) (Fig S3). Part of intracellular P neutralized by counter cations (K^+^ and Mg^2+^) was emitted in to the environment [33, 34], while only P transporters (the high affinity *pst* and low affinity *pit*) in Clade IIA displayed moderate expression.

The Gly module could directly supply 2 ATP molecules per glycogen molecule (level III). Other energy pools, such as AN ETP (level III) were activated for both Types. The Complete TCA Cycle in Clade IB also supported extra energy in the form of GTP. However, it did not suggest that the energy supplied by PolyP was insufficient for the intensive consumption of Accumulibacter, the activity of Gly module and AN ETP were more likely to be regulated for carbon providing and redox homeostasis maintaining.

Clade IB could be provided with limited phosphorus, judging from the low expression of PolyP module, *pst* and *pit*, when additional energy was compensated by the high expression of AN ETP coupling with nitrate reduction, Complete and Partial TCA Cycles. With limited extracellular phosphorus in the specific EBPR reactor of Clade IB [18], the accumulation of phosphorus in intracellular PolyP module was insufficient to provide enough electrochemical gradient and the energy supply for the activity of phosphorus transporters (*pst* and *pit*).

As clarified before, Accumulibacter mainly invest energy in the activation of acetate and Pro (level II-III) and the ABC transporters. In addition, proton (*pntA/B*) and Na^+^ (*hppA*) pumps were actively expressed in both Types (level II-III) to generate PMF using ATP and *PPi*. The Calvin Cycle also consumed ATP for carbon fixation. Other energy-consuming modules in Clade IIA included the oxidative branch of Split TCA Cycle (requiring GTP, level III) and S module (assimilatory sulfate reduction, level III).

*AE Carbon flow*

Both two Clades shared a consistent expression profile in AE phase.

The primary carbon source in AE period was donated by the intracellular PHA granules with an active expression pattern for both Types (level III). Besides the PHA module, the slight activity of Calvin Cycle of two Types could also supply additional carbon source flowing to Acyl-CoA through 3-PG, which was incentive for Accumulibacter during carbon famine period. Although it was predicted to be sensitive to DO, the *rbcL* rubisco was barely changed during the whole EBPR cycle. The expression of the sharing pathways of Calvin Cycle and Gly module were mainly contributed by the glycolysis and gluconeogenesis. Furthermore, the *actP* transporter was activated (level III) to import the remaining acetate and Pro. Their conversion to Acyl-CoA and Pro-CoA was fulfilled by both high affinity (*acs* and *prpE*) and low affinity (*ackA* and *pta*) enzymes (level II-III). We were intrigued about the dramatically increase of *actP*, *ackA* and *pta* in Clade IB from moderate expression in AN phase to high expression in AE phase. One possible explanation might be that the *actP* and low affinity pathway could be more active during extracellular carbon famine phase [21, 32]. In AN phase, both of them displayed an increasing trend in Clade IIA (Fig S5) responding to the decreasing concentration of available acetate. The delay of transcriptional increase in Clade IB could be explained by the hypothesis that Clade IB was supplied with adequate extracellular carbon sources that were not exhausted until the AE phase.

Gly and Complete TCA Cycle were proposed to be the major carbon consumers in AE phase, which were all highly expressed (II-III) in both Types. As discussed in previous section, Partial TCA Cycle, as an intermediate module, may coexist with Complete TCA Cycle to reclaim carbon source for glycogen replenishment. The *fnr* regulator for Partial TCA shunt was also activated (III). Another carbon flow to Gly module was the direct conversion of Acyl-CoA to Pyr with an active expression level III. The possible limiting steps of Gly module were the conversion from Pyr to phosphoenolpyruvate (PEP) (*oadB* and *ppc*). Both the Partial TCA Cycle and Gly modules in Clade IB displayed a more modest expression than Clade IIA. Even though it could not directly infer the ability of glycogen storage in Type I Accumulibacter, it could partially elucidate the inadequate reducing power produced by Gly module in AN phase. However, further investigations are required to detect the activity of related enzymes in Gly module and the production of electrons by metaproteomics analysis and fluorescent sensor based guanidinium-tethered tetraphenylethene (TPE).

Pro module was also actively expressed in both Types to allocate Pro-CoA from PHA module. This was unexpected for Clade IB since almost none Pro-CoA were generated from known sources in AN phase. It was also surprising that the carbon from Pro-CoA was flowing both into TCA Cycle (III) and to Acyl-CoA (II) even with the acetate famine in AE phase. Further metaproteomic data is required to confirm the activity of these enzymes. The supplementary carbon consumers for cell growth and reproduction were consistently expressed in two Types, such as N (glutamate synthesis, III), LCFA (II-III), AA (I-II), EPS (I-II) and PL (I) modules.

*AE Electron flow*

The majority of reducing equivalents for two Types was supported by Complete TCA Cycle in the form of NADH, QH_2_ and FADH_2_ with an active expression at level III (Fig S3). Partial TCA Cycle (I-II, NADH) and PHA module (level III, NADPH) also supplied additional electrons, while their major function was involved in the carbon and energy flows.

Electrons were invested primarily in AE ETP for energy production. The activity of C.I, C.II, C.III and two ATPases were highly expressed coupling with Complete TCA Cycle to balance reducing equivalents. The Complex IV (C.IV) cytochrome c oxidase using O_2_ as TEAs was revealed as the limiting step for Clade IB (level II) judged only by its relative cellular expression level. The other electron-consuming modules included Gly (level III, NADH), LCFA (level II-III, NADH, NADPH and FADH), N (level II-III, NADH and NADPH), S (assimilatory sulfate reduction, level III, NADPH), Calvin Cycle (level I-II, NADPH) and EPS (level I-II, NADH) modules and the transformation of Acyl-CoA to Pyr (level III, NADH and fdH_2_). The consuming of NADH and NADPH by hydrogenases was also detected (level III) for both Types during AE phase, which might indicate that the reducing equivalents were excessive for energy generation and cell growth. It was also supported by the active denitrification and dissimilatory nitrogen reduction in AE phase to balance reducing power. Another possible cause would be the accumulation of NADH due to the insufficient efficiency of transhydrogenases. Similar to AN phase, the interconversion of NADH to fdH_2_ by *fpr* (III) was consistent with the Acyl-CoA to Pyr transformation and the NADPH and NADH interconversion by *pntAB* was actively expressed at level II-III in both Types.

*AE Energy flow*

As discussed previously, the energy in AE phase was primarily generated by AE ETP in both Types. The Complete TCA also provided energy in the form of GTP at high expression level III. When limited extracellular phosphorus is provided, Partial TCA Cycle should be provoked by Accumulibacter for additional glycogen replenishment to fuel AN ETP in the following AN phase as extra energy supplier in addition to PolyP [35].

The most primary electron sink in AE phase was PolyP module consuming ATP (III) and a few GTP (II-III). The related transporters *pst* (level I-III) and *pit* (level III) were activated to import P. Other electron-consuming modules, such as Gly, LCFA, N, EPS and Calvin Cycle were dominated mainly by electron and carbon flows. Nitrogen fixation, as an energy expensive process, was scarcely expressed as level 0-I during both two phases, mainly because of its little beneficial to Accumulibacter in ammonia feast environments. The expression of cation pumps (*pntA/B* and *hppA*) was activated at level III. The electrochemical potential-driven transporters such as *kup, actP* and *pit* displayed the same trend as cation pumps (Fig S5), driven by PMF. However, other factors such as the co-expression (*actP* and *acs*) and regulatory systems could also influence the expression of these transporters**.**

*Correspondence of modules*

Besides the correspondence inside each flow, this study addressed a new aspect to integrate separated flow into **one** well-cooperated system. Obviously, the main upstream of carbon flow (PHA, acetate and Pro) influences the availability of carbon sources, which consequently affects the carbon flow to carbon consumers. In AE phase, reducing power is released from both the primary carbon-providing module (PHA) and carbon-consuming module (Complete TCA Cycle), which implies that the upstream of electron flow corresponds to the carbon flow. As utilized by electron-consuming modules (mostly carbon-consuming modules) and energy-producing ETP, the electron flow further affects the downstream of energy and carbon flow by electrons availability and the upstream of energy flow by changing redox status as coupling reactions. To be specific, PHA module exhibited the expression trend of an increase at AE beginning and a decrease at AE ending (Fig S5). Accordingly, Complete and Partial TCA Cycles, as the primary electron producers, displayed the same trend as PHA module. The other carbon-consuming modules were driven by both the carbon and electrons, consistently corresponding to PHA module and TCA Cycle. The correlation between energy and electron flow was demonstrated by the same trend of AE ETP expression. These observations additionally emphasized on the importance of reducing equivalents to interconnect among carbon, electron and energy flow to comprehensively analyze the correspondence of different modules as one entire system.

**Determining the flow direction of reversible modules**

The direction of reversible modules is crucial to determine their roles in three main flows (carbon, electron and energy) and the cell condition of Accumulibacter. Here, we demostrated a convincing method by considering both the essential pathways and the coordination among modules in carbon and electron flows.

To determine the flow direction of reversible modules, such as the TCA Cycle and LCFA, the activity of the essential pathways/genes in carbon flow were firstly considered, based on the known knowledge of Accumulibacter functions. For example, to differentiate the three modes of TCA Cycle (Complete, Partial and Split TCA Cycles), three essential aspects of pathways/genes for consideration include the glyoxylate shunt of Partial TCA Cycle, two genes to branch succinyl-CoA (methylmalonyl-CoA mutase *mut* to Pro module against the oxidoreductase *korAB* in TCA Cycle), and the transcriptional difference between the left and right arms [22] in TCA Cycle.

For Clade IB, no expression of the *mut* gene for converting Pro-CoA through succinyl-CoA was detected [18] while the high expression of the other two aspects of pathways/genes were observed, implying that Clade IB employed Partial TCA Cycle (glyoxylate shunt flowing to oxaloacetate) and Complete TCA Cycle (decarboxylation steps) instead of Split TCA Cycle. For Clade IIA, however, it showed almost completely different behaviors. The high expression of *mut* in Split TCA Cycle and methylmalonyl-CoA decarboxylase *mmcD* in Pro module compared with the low expression of both glyoxylate shunt and initial left arm of TCA Cycle implicated the dominant mode of TCA Cycle was the oxidative branch of Split TCA Cycle in Clade IIA (Fig S3).

However, in principle, the direction (forward or reverse) in TCA Cycle was more likely to be affected by intracellular redox condition rather than carbon source availability. Therefore, the above observation revealed by carbon flow was additionally validated by the balance between carbon and electron flows in both Clades. For Clade IB, the Complete and Partial TCA Cycles served as supplementary electron providing modules when the electrons provided by Gly were limited compared with the overloaded extracellular acetate under that experimental condition [18]. On the contrary, the oxidative branch of Split TCA Cycle coordinated with other electron-consuming modules (hydrogenases, sulfite reduction, dissimilatory nitrite reduction and denitrification) to balance excessive electrons from Gly module compared with limited acetate under that experimental condition [19].

**Supplementary Tables**

Table S1. Relationship between the coverage, accuracy, FN and FP of core-, dispensable- and strain-specific- genomes (FN1=FP2, FN2=FP3, FN3=$max[P\left( \bar{G}i \right)]$).

|  | Core-genome | Dispensable-genome | Strain-specific-genome |
| --- | --- | --- | --- |
| Coverage | 100%-FN1 | 100%-FN2 | 100%-FN3 |
| Accuracy | \^*^ | 100%-FP2 | 100%-FP3 |

^*^Because that the incompleteness part of genomes is unknown and unpredictable, the accuracy/FP1 of core-genome was unable to be calculated.

Table S2. The estimated completeness, contamination and accession number of 13 available Accumulibacter draft genomes.

| Genomes | Completeness | Contamination | Accession number |
| --- | --- | --- | --- |
| AccIA_1 | 0.99 | 0.00 | GCA_000585075.1 |
| AccIA_2 | 0.88 | 0.00 | IMG 2687453699 |
| AccIB | 0.9 | 0.03 | GCA_000987445.1 |
| AccIC | 0.93 | 0.01 | GCA_000585055.1 |
| AccIIA_1 | 1.00 | 0.00 | GCA_000024165.1 |
| AccIIA_2 | 1.00 | 0.06 | GCA_900089955.1 |
| AccIIC_1 | 0.98 | 0.02 | GCA_000584975.2 |
| AccIIC_2 | 0.94 | 0.01 | GCA_000584955.2 |
| AccIIC_3 | 0.92 | 0.03 | GCA_000987395.1 |
| AccIIC_4 | 0.82 | 0.03 | GCA_000585035.2 |
| AccIIF_1 | 0.93 | 0.00 | GCA_000585015.1 |
| AccIIF_2 | 0.77 | 0.00 | GCA_000584995.1 |
| AccIIF_3 | 0.79 | 0.08 | GCA_000585095.1 |

Table S3. The raw reads and normalized metatranscriptomic data as RPKM, MRPKM, CRPKM and LCRPKM for Clades IB and IIA.

| Table S4. The estimated FN and FP rates of core-, dispensable- and strain-specific-genomes with different cutoff from 1 to 13. | | |
| --- | --- | --- |
| Cutoff for a core gene | Core FN rate  (Dispensable FP rate) | Dispensable FN rate (Strain-specific FP rate) |
| 13 | 7.15E-01 | \ |
| 12 | 3.24E-01 | 1.61E-13 |
| 11 | 9.48E-02 | 6.41E-12 |
| 10 | 1.84E-02 | 1.82E-10 |
| 9 | 2.40E-03 | 4.01E-09 |
| 8 | 2.14E-04 | 7.36E-08 |
| 7 | 1.28E-05 | 1.16E-06 |
| 6 | 4.97E-07 | 1.61E-05 |
| 5 | 1.19E-08 | 1.98E-04 |
| 4 | 1.60E-10 | 2.16E-03 |
| 3 | 1.04E-12 | 2.06E-02 |
| 2 | 5.33E-15 | 1.63E-01 |
| 1 | 0.00E+00 | \ |

Table S5. Material and energy flow (electron, energy and carbon) of each module in anaerobic (AN) compared to aerobic (AE) phase of an EBPR biochemical cycle. Production, consumption of material and reaction potential for both directions in one phase were highlighted in green, red and yellow respectively. The abbreviations of modules and chemical components are the listed in Fig 2.

Table S6. Providers and consumers of electron, energy and carbon in anaerobic (AN) and aerobic (AE) phases of an EBPR biochemical cycle. Primary consumers or providers were highlighted in bold. The abbreviations of modules and chemical components are the listed in Fig 2.

**Supplementary Figures**


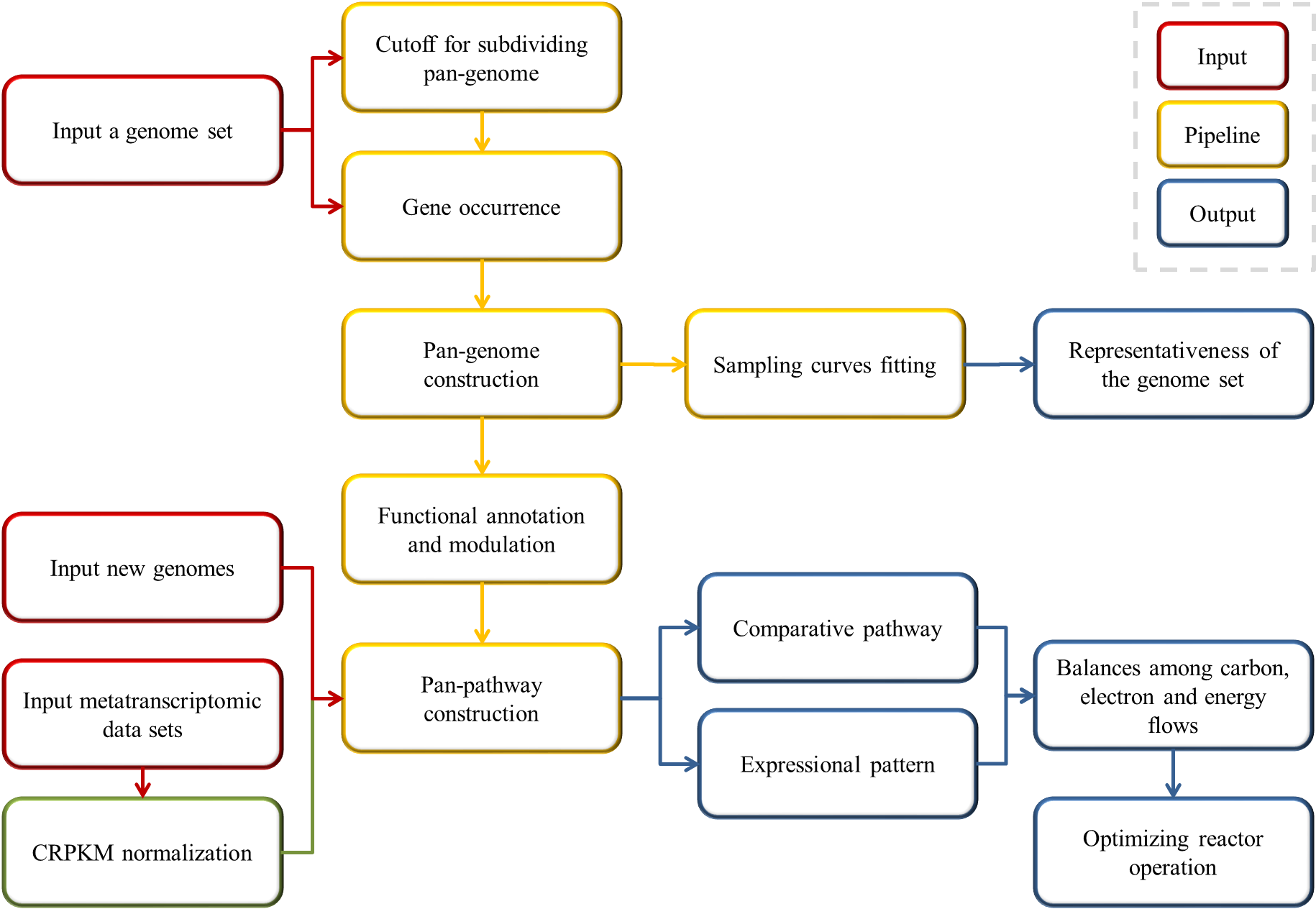
Fig S1. The Technical flow of this study.


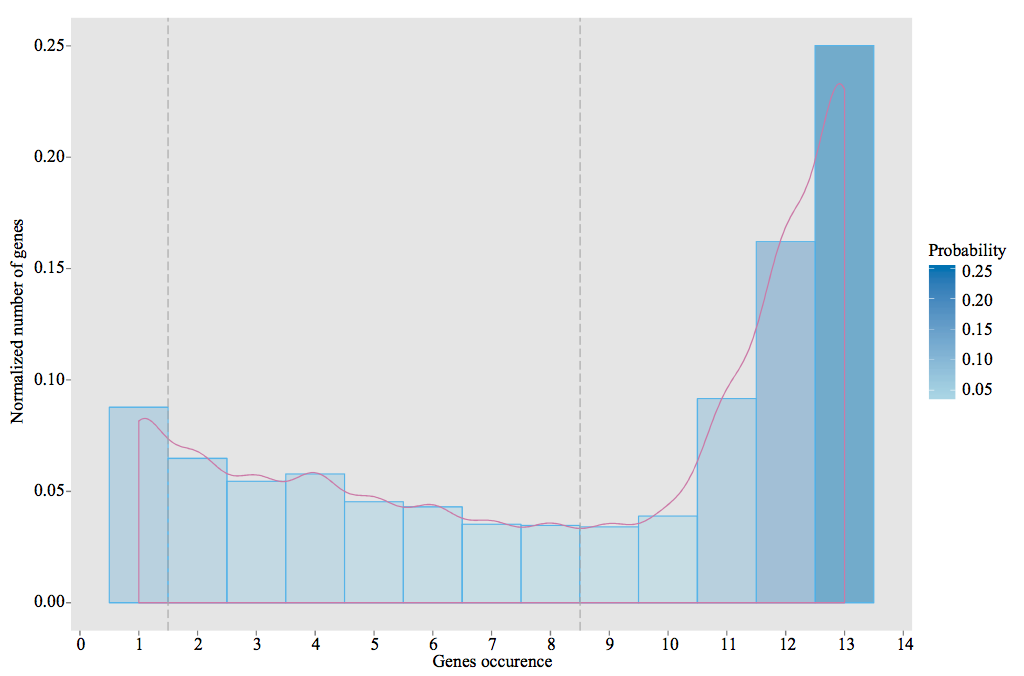


Fig S2. A density curve showing the distribution of the occurrence frequency of genes in the Accumulibacter pan-genome, determined by the integrated alignment results. Each bar represented the normalized number of genes (number of genes against the total number of filtered genes) within the same indicated occurrence. The cluster with occurrence in one genome (itself) represents genes with no homologs, whereas the cluster with the at least 8 occurrence compromised the core-genome pool.


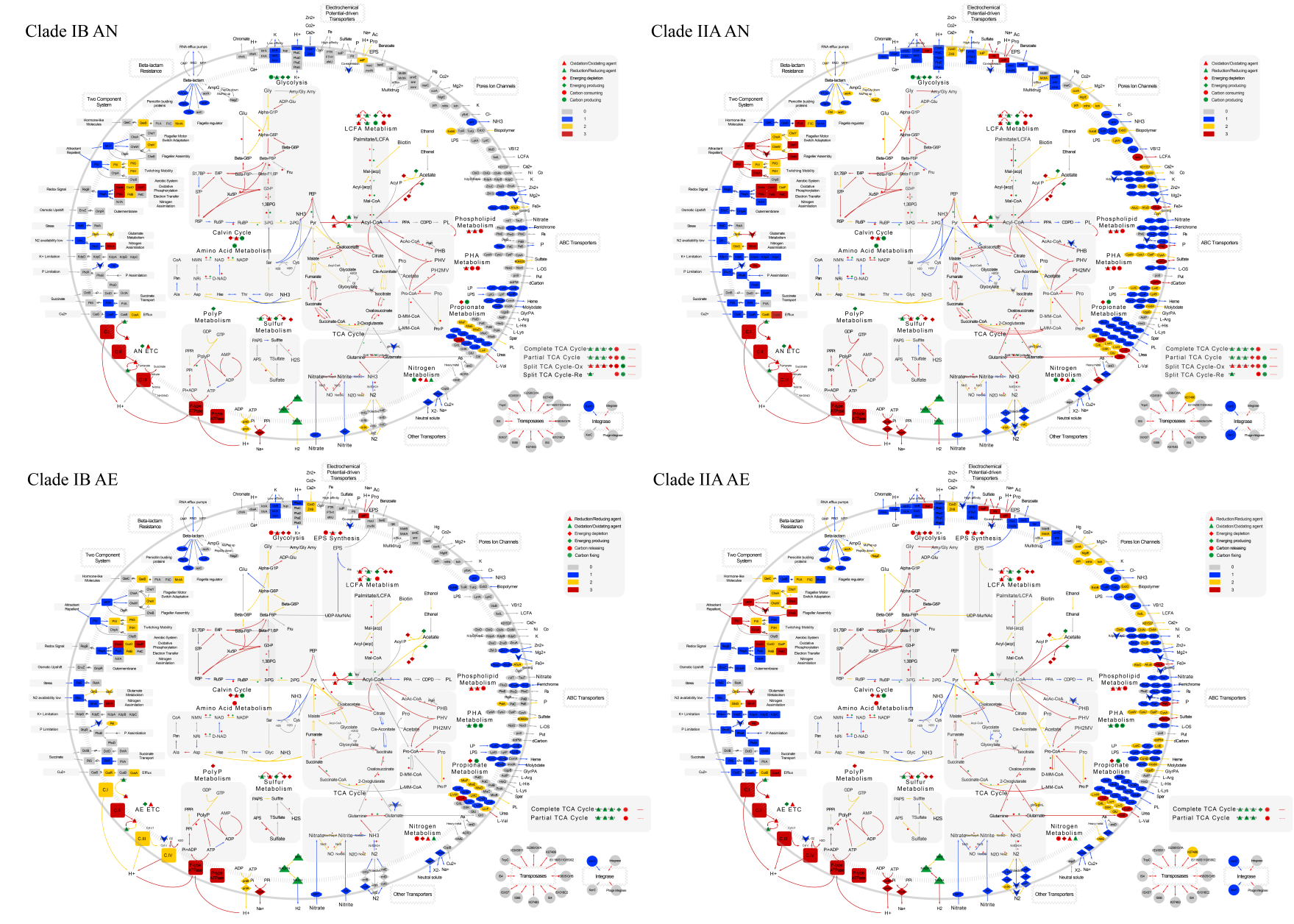


Fig S3. The comparison of RNA expression of Type I and Type II Accumulibacter in anaerobic and aerobic phases. The abbreviations of modules and chemical components are the listed in Fig 2.


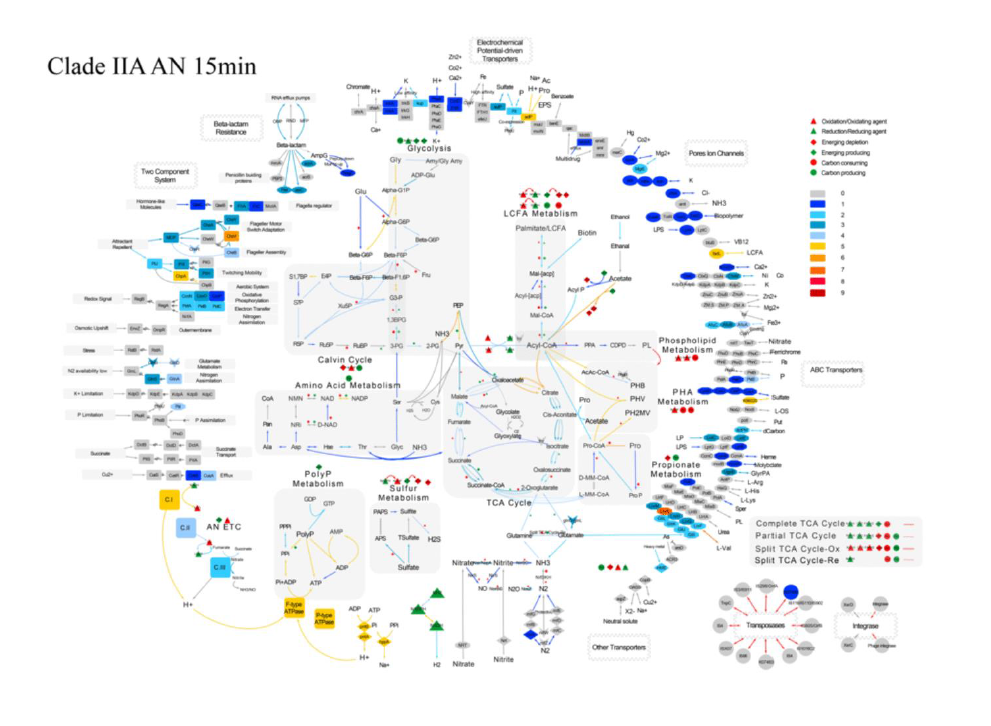


Fig S4. The dynamic pattern of RNA expression of Clade IIA highlighted in the constructed Accumulibacter Pan-genome pathway. Abbreviations: AN, anaerobic phase; AE, aerobic phase.


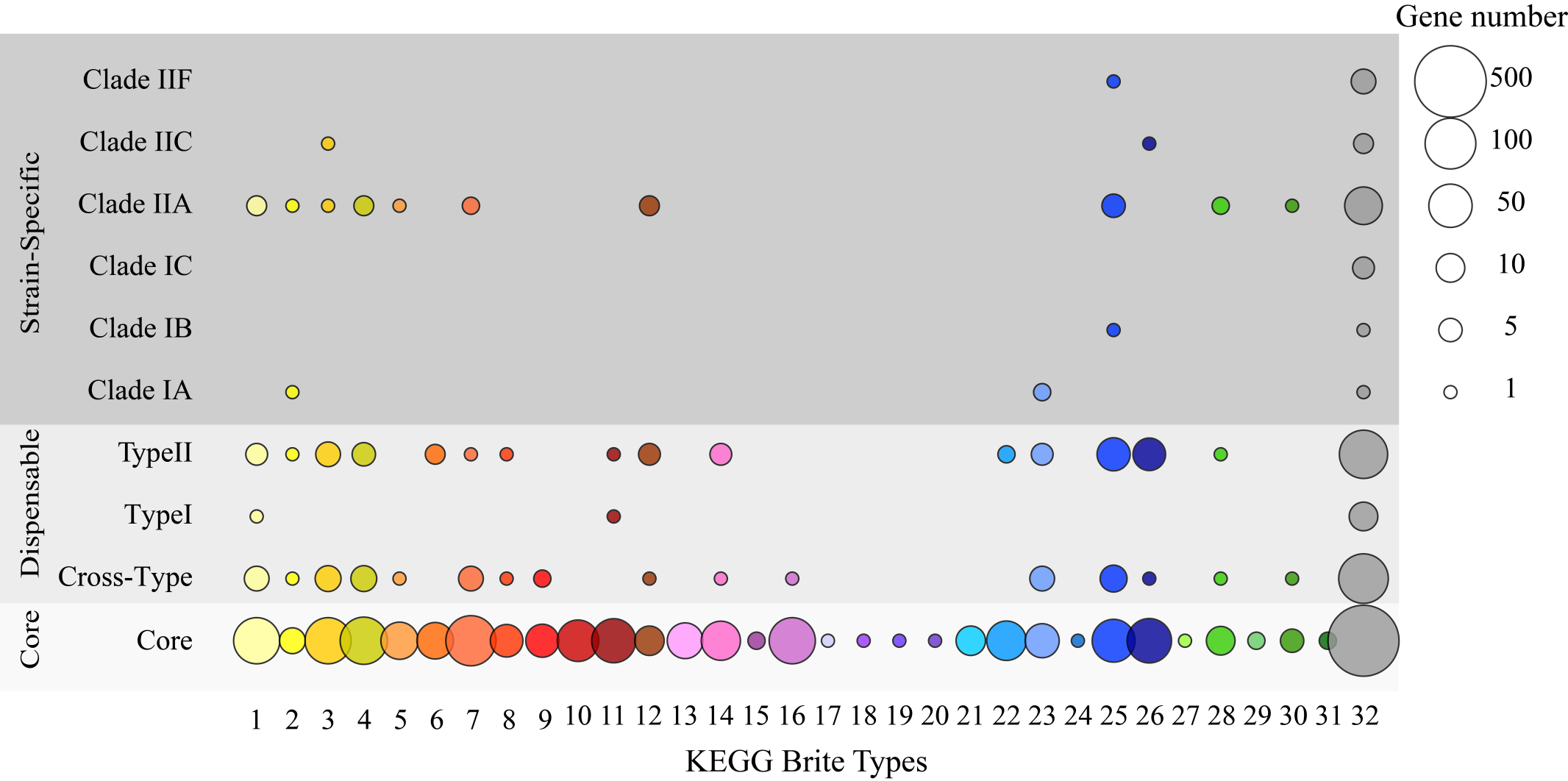


Fig S5. The distribution of KEGG function types (brite types) of all non-redundant genes/KOs in Accumulibacter pan-pathway (core-, dispensable- and strain-specific pathways). The function of corresponding KEGG brite type: metabolism of yellow and red colors (1, amino acid metabolism; 2, biosynthesis of other secondary metabolites; 3, carbohydrate metabolism; 4, energy metabolism; 5, glycan biosynthesis and metabolism; 6, lipid metabolism; 7, metabolism of cofactors and vitamins; 8, metabolism of other amino acids; 9, metabolism of terpenoids and polyketides; 10, nucleotide metabolism; 11, overview; 12, xenobiotics biodegradation and metabolism); genetic information processing of pink color (13, folding, sorting and degradation; 14, replication and repair; 15, transcription; 16, translation); organismal systems of purple color (17, aging; 18, environmental adaptation; 19, immune system; 20, nervous system); cellular processes of light blue color (21, cell growth and death; 22, cell motility; 23, cellular community - prokaryotes; 24, transport and catabolism); environmental information processing of dark blue color (25, membrane transport; 26, signal transduction); human diseases of green color (27, cancers; 28, drug resistance; 29, endocrine and metabolic diseases; 30, infectious diseases; 31, neurodegenerative diseases); 32, none of grey color.

Reference

1. Meric G, Yahara K, Mageiros L, Pascoe B, Maiden MC, Jolley KA, Sheppard SK: **A reference pan-genome approach to comparative bacterial genomics: identification of novel epidemiological markers in pathogenic Campylobacter.** *PLoS One* 2014, **9:**e92798.

2. Tettelin H, Masignani V, Cieslewicz MJ, Donati C, Medini D, Ward NL, Angiuoli SV, Crabtree J, Jones AL, Durkin AS, et al: **Genome analysis of multiple pathogenic isolates of Streptococcus agalactiae: implications for the microbial "pan-genome".** *Proc Natl Acad Sci U S A* 2005, **102:**13950-13955.

3. Mongodin EF, Casjens SR, Bruno JF, Xu Y, Drabek EF, Riley DR, Cantarel BL, Pagan PE, Hernandez YA, Vargas LC: **Inter-and intra-specific pan-genomes of Borrelia burgdorferi sensu lato: genome stability and adaptive radiation.** *BMC genomics* 2013, **14:**1.

4. Camacho C, Coulouris G, Avagyan V, Ma N, Papadopoulos J, Bealer K, Madden TL: **BLAST+: architecture and applications.** *BMC Bioinformatics* 2009, **10:**421.

5. Lapierre P, Gogarten JP: **Estimating the size of the bacterial pan-genome.** *Trends Genet* 2009, **25:**107-110.

6. Kweon O, Kim SJ, Blom J, Kim SK, Kim BS, Baek DH, Park SI, Sutherland JB, Cerniglia CE: **Comparative functional pan-genome analyses to build connections between genomic dynamics and phenotypic evolution in polycyclic aromatic hydrocarbon metabolism in the genus Mycobacterium.** *BMC Evol Biol* 2015, **15:**21.

7. Tettelin H, Riley D, Cattuto C, Medini D: **Comparative genomics: the bacterial pan-genome.** *Current opinion in microbiology* 2008, **11:**472-477.

8. Ahmed A, Earl J, Retchless A, Hillier SL, Rabe LK, Cherpes TL, Powell E, Janto B, Eutsey R, Hiller NL, et al: **Comparative genomic analyses of 17 clinical isolates of Gardnerella vaginalis provide evidence of multiple genetically isolated clades consistent with subspeciation into genovars.** *J Bacteriol* 2012, **194:**3922-3937.

9. Vernikos G, Medini D, Riley DR, Tettelin H: **Ten years of pan-genome analyses.** *Curr Opin Microbiol* 2015, **23:**148-154.

10. Kanehisa M, Sato Y, Kawashima M, Furumichi M, Tanabe M: **KEGG as a reference resource for gene and protein annotation.** *Nucleic Acids Res* 2016, **44:**D457-462.

11. Huerta-Cepas J, Szklarczyk D, Forslund K, Cook H, Heller D, Walter MC, Rattei T, Mende DR, Sunagawa S, Kuhn M, et al: **eggNOG 4.5: a hierarchical orthology framework with improved functional annotations for eukaryotic, prokaryotic and viral sequences.** *Nucleic Acids Res* 2016, **44:**D286-293.

12. Galperin MY, Makarova KS, Wolf YI, Koonin EV: **Expanded microbial genome coverage and improved protein family annotation in the COG database.** *Nucleic Acids Res* 2015, **43:**D261-269.

13. Shannon P, Markiel A, Ozier O, Baliga NS, Wang JT, Ramage D, Amin N, Schwikowski B, Ideker T: **Cytoscape: a software environment for integrated models of biomolecular interaction networks.** *Genome research* 2003, **13:**2498-2504.

14. Nishida K, Ono K, Kanaya S, Takahashi K: **KEGGscape: a Cytoscape app for pathway data integration.** *F1000Research* 2014, **3**.

15. Xia Y, Wang Y, Wang Y, Chin FY, Zhang T: **Cellular adhesiveness and cellulolytic capacity in Anaerolineae revealed by omics-based genome interpretation.** *Biotechnol Biofuels* 2016, **9:**111.

16. Schirrmeister BE, Dalquen DA, Anisimova M, Bagheri HC: **Gene copy number variation and its significance in cyanobacterial phylogeny.** *BMC microbiology* 2012, **12:**177.

17. Handley KM, Bartels D, O'Loughlin EJ, Williams KH, Trimble WL, Skinner K, Gilbert JA, Desai N, Glass EM, Paczian T, et al: **The complete genome sequence for putative H2- and S-oxidizer Candidatus Sulfuricurvum sp., assembled de novo from an aquifer-derived metagenome.** *Environmental Microbiology* 2014, **16:**3443-3462.

18. Mao Y, Yu K, Xia Y, Chao Y, Zhang T: **Genome reconstruction and gene expression of "Candidatus Accumulibacter phosphatis" Clade IB performing biological phosphorus removal.** *Environ Sci Technol* 2014, **48:**10363-10371.

19. Oyserman BO, Noguera DR, Del Rio TG, Tringe SG, McMahon KD: **Metatranscriptomic insights on gene expression and regulatory controls in Candidatus Accumulibacter phosphatis.** *ISME J* 2015.

20. Wilmes P, Andersson AF, Lefsrud MG, Wexler M, Shah M, Zhang B, Hettich RL, Bond PL, VerBerkmoes NC, Banfield JF: **Community proteogenomics highlights microbial strain-variant protein expression within activated sludge performing enhanced biological phosphorus removal.** *ISME J* 2008, **2:**853-864.

21. Martin HG, Ivanova N, Kunin V, Warnecke F, Barry KW, McHardy AC, Yeates C, He S, Salamov AA, Szeto E, et al: **Metagenomic analysis of two enhanced biological phosphorus removal (EBPR) sludge communities.** *Nat Biotechnol* 2006, **24:**1263-1269.

22. He S, McMahon KD: **'Candidatus Accumulibacter' gene expression in response to dynamic EBPR conditions.** *ISME J* 2011, **5:**329-340.

23. Burow LC, Mabbett AN, Blackall LL: **Anaerobic glyoxylate cycle activity during simultaneous utilization of glycogen and acetate in uncultured Accumulibacter enriched in enhanced biological phosphorus removal communities.** *ISME J* 2008, **2:**1040-1051.

24. Zhou Y, Pijuan M, Zeng RJ, Yuan Z: **Involvement of the TCA cycle in the anaerobic metabolism of polyphosphate accumulating organisms (PAOs).** *Water Res* 2009, **43:**1330-1340.

25. Wexler M, Richardson DJ, Bond PL: **Radiolabelled proteomics to determine differential functioning of Accumulibacter during the anaerobic and aerobic phases of a bioreactor operating for enhanced biological phosphorus removal.** *Environ Microbiol* 2009, **11:**3029-3044.

26. He S, Kunin V, Haynes M, Martin HG, Ivanova N, Rohwer F, Hugenholtz P, McMahon KD: **Metatranscriptomic array analysis of 'Candidatus Accumulibacter phosphatis'-enriched enhanced biological phosphorus removal sludge.** *Environ Microbiol* 2010, **12:**1205-1217.

27. Jendrossek D: **Polyhydroxyalkanoate granules are complex subcellular organelles (carbonosomes).** *Journal of bacteriology* 2009, **191:**3195-3202.

28. Lin X, Handley KM, Gilbert JA, Kostka JE: **Metabolic potential of fatty acid oxidation and anaerobic respiration by abundant members of Thaumarchaeota and Thermoplasmata in deep anoxic peat.** *ISME J* 2015, **9:**2740-2744.

29. Oehmen A, Lemos PC, Carvalho G, Yuan Z, Keller J, Blackall LL, Reis MA: **Advances in enhanced biological phosphorus removal: from micro to macro scale.** *Water Res* 2007, **41:**2271-2300.

30. Seviour RJ, Mino T, Onuki M: **The microbiology of biological phosphorus removal in activated sludge systems.** *FEMS Microbiology Reviews* 2003, **27:**99-127.

31. He S, McMahon KD: **Microbiology of 'Candidatus Accumulibacter' in activated sludge.** *Microb Biotechnol* 2011, **4:**603-619.

32. Hesselmann R, Von Rummell R, Resnick SM, Hany R, Zehnder A: **Anaerobic metabolism of bacteria performing enhanced biological phosphate removal.** *Water Research* 2000, **34:**3487-3494.

33. Tykesson E, Blackall L, Kong Y, Nielsen PH, la Cour Jansen J: **Applicability of experience from laboratory reactors with biological phosphorus removal in full-scale plants.** *Water science and technology* 2006, **54:**267-275.

34. Schönborn C, Bauer H-D, Röske I: **Stability of enhanced biological phosphorus removal and composition of polyphosphate granules.** *Water research* 2001, **35:**3190-3196.

35. Zhou Y, Pijuan M, Zeng RJ, Lu H, Yuan Z: **Could polyphosphate-accumulating organisms (PAOs) be glycogen-accumulating organisms (GAOs)?** *Water Res* 2008, **42:**2361-2368.
