# Supplementary material for: Mining traits for the enrichment and isolation of not-yet-cultured populations
Source: Microbiome. 2019 Jun 25;7:96. doi: 10.1186/s40168-019-0708-4 (PMC6593511; doi:10.1186/s40168-019-0708-4)
Supplement: Supplementary file 3 — Table S5. Material and energy flow (electron, energy, and carbon) of each module in anaerobic (AN) compared to aerobic (AE) phase of an EBPR biochemical cycle. Production, consumption of material, and reaction potential for both directions in one phase were highlighted in green, red, and yellow, respectively. The abbreviations of modules and chemical components are the listed in Fig. 2. (DOCX 19 kb) [file 40168_2019_708_MOESM3_ESM.docx]

| Table S5. Material and energy flow (electron, energy and carbon) of each module in anaerobic (AN) compared to aerobic (AE) phase of an EBPR biochemical cycle. Production, consumption of material and reaction potential for both directions in one phase were highlighted in green, red and yellow respectively. The abbreviations of modules and chemical components are the listed in Fig 2. | | | | | | | | | | |
| --- | --- | --- | --- | --- | --- | --- | --- | --- | --- | --- |
| **Phases** | **AN Phase** | | | | | **AE Phase** | | | | |
| Flow | Electron | Energy | | Carbon | | Electron | Energy | | Carbon | |
| Gly Module | NADH | ATP GTP | | Pyr | | NADH | ATP GTP | | Pyr | |
| Complete TCA Cycle | NADH QH_2_ FADH_2_ | GTP | | Acetate | | NADH QH_2_ FADH_2_ | GTP | | Acetate | |
| Partial TCA Cycle | NADH QH_2_ FADH_2_ | GTP | | Acetate | Pro Oxaloacetate | NADH QH_2_ FADH_2_ |  | | Acetate | Oxaloacetate |
| Split TCA Cycle-Ox | NADH QH_2_ FADH_2_ | GTP | | Acetate | Pro | **\** | **\** | | **\** | |
| Split TCA Cycle-Re | NADH |  | | Acetate | Pro | **\** | **\** | | **\** | |
| Pyr <> Acyl-CoA | fdH_2_ NADH |  | | Pyr | Acetate | fdH2 NADH |  | | Acetate | Pyr |
| Acetate > Acyl-CoA |  | ATP | AMP | Acetate | |  | ATP | AMP | Acetate | |
| Calvin Cycle | NADPH | ATP | | 3-PG | | NADPH | ATP | | 3-PG | |
| PHA Module | NADPH |  | | Acetate Pro | | NADPH |  | | Acetate Pro | |
| LCFA Module | NAD(P)H FADH | ATP AMP | | Acetate | | NAD(P)H FADH | ATP | AMP | Acetate | |
| PL Module | NAD(P)H |  | | Acetate | | NAD(P)H |  | | Acetate | |
| Pro Module |  | ATP | | Pro | |  | ATP | | Pro | |
| AA Module |  |  | | Pyr | |  |  | | Pyr | |
| N Module | NAD(P)H | ATP | | Pro | | NAD(P)H | ATP | | Acetate | |
| S Module | NADPH | ATP | AMP |  | | NADPH | ATP | AMP |  | |
| P Module |  | ATP | |  | |  | ATP | |  | |
| EPS Module | **\** | **\** | | **\** | | NADH | ATP | | Beta-F6P | |
| ETP | NADH | ATP | |  | | NADH | ATP | |  | |
