# Supplementary material for: Mining traits for the enrichment and isolation of not-yet-cultured populations
Source: Microbiome. 2019 Jun 25;7:96. doi: 10.1186/s40168-019-0708-4 (PMC6593511; doi:10.1186/s40168-019-0708-4)
Supplement: Supplementary file 4 — Table S6. Providers and consumers of electron, energy, and carbon in anaerobic (AN) and aerobic (AE) phases of an EBPR biochemical cycle. Primary consumers or providers were highlighted in bold. The abbreviations of modules and chemical components are the listed in Fig. 2. (DOCX 17 kb) [file 40168_2019_708_MOESM4_ESM.docx]

| Table S6. Providers and consumers of electron, energy and carbon in anaerobic (AN) and aerobic (AE) phases of an EBPR biochemical cycle. Primary consumers or providers were highlighted in bold. The abbreviations of modules and chemical components are the listed in Fig 2. | | |
| --- | --- | --- |
| **Phases** | **AN Phase** | **AE Phase** |
| Carbon pool | **Acetate or Pro**, Gly, LCFA, AA, N (Glutamine/Glutamate), Calvin Cycle (AN Ending) | **PHA**, Calvin Cycle (AE Beginning), Acetate > Acyl-CoA, Pro |
| Electron pool | **Gly**, Pyr > Acyl-CoA, Complete TCA Cycle, Partial TCA Cycle, Split TCA Cycle (reductive branch), N, LCFA | **Complete TCA Cycle**, PHA, Partial TCA Cycle |
| Energy pool | **PolyP**, AN ETP, Gly, Complete TCA Cycle, LCFA | **AE ETP**, **Complete TCA Cycle** |
| Carbon consumer | **PHA**, Complete TCA Cycle, PL, LCFA | **Complete TCA Cycle**, **Gly**, LCFA, PL, AA, N, EPS |
| Electron consumer | **PHA**, Split TCA Cycle (oxidative branch), PL, N, LCFA, S, AN ETP, Calvin Cycle (AN Ending), Hydrogenases | **AE ETP**, **Gly**, Acyl-CoA > Pyr, LCFA, PL, EPS, S, Calvin Cycle, Hydrogenases |
| Energy consumer | **Acetate > Acyl-CoA**, **Pro > Pro-CoA**, ABC Transporters, N, LCFA, S, Split TCA Cycle (oxidative branch), Calvin Cycle (AN Ending) | **PolyP**, Gly, Pro, Acetate > Acyl-CoA, Pro > Pro-CoA, LCFA, EPS, S, Calvin Cycle, N |
